# Supplementary material for: Trajectory of skill acquisition, loss, and regain in females with classic Rett syndrome
Source: J Neurodev Disord. 2026 Mar 12;18:20. doi: 10.1186/s11689-026-09680-6 (PMC13094048; doi:10.1186/s11689-026-09680-6)
Supplement: Supplementary file 1 — Supplementary Material 1 [file 11689_2026_9680_MOESM1_ESM.pdf]

Table S1: Demographic Information

Race	Ethnicity N (%)			Total N (%)
	Not Hispanic	Hispanic	Refused/Unknown	
**White**	875 (71.3)	130 (10.6)	16 (1.3)	1021 (83.1)
**Black**	51 (4.2)	3 (0.2)	-	54 (4.4)
**Asian**	41 (3.3)	3 (0.2)	1 (0.08)	45 (3.7)
**Native American**	2 (0.16)	5 (0.4)	-	7 (0.6)
**Pacific Islander/Hawaiian**	1 (0.08)	-	-	1 (0.1)
**Multiple/Mixed**	55 (4.5)	17 (1.4)	1 (0.08)	73 (5.9)
**Refused/Unknown**	2 (0.16)	24 (2.0)	1 (0.08)	27 (2.2)
**Total**	1027 (83.7)	182 (14.8)	19 (1.5)	1228 (100)
