# Supplementary material for: Trajectory of skill acquisition, loss, and regain in females with classic Rett syndrome
Source: J Neurodev Disord. 2026 Mar 12;18:20. doi: 10.1186/s11689-026-09680-6 (PMC13094048; doi:10.1186/s11689-026-09680-6)
Supplement: Supplementary file 2 — Supplementary Material 2 [file 11689_2026_9680_MOESM2_ESM.pdf]

Table S2 *MECP2* Mutation Frequency

Mutation Group	N (%)
R106W	47 (3.8)
R133C	77 (6.3)
T158M	131 (10.7)
R168X	134 (10.9)
R255X	118 (9.6)
R270X	76 (6.2)
R294X	78 (6.4)
R306C	100 (8.1)
C-terminal Truncations	137 (11.2)
Large Deletions	111 (9.0)
Early Truncations	135 (11.0)
Other Point Mutations	84 (6.8)
All	1228 (100)
