# Supplementary material for: Trajectory of skill acquisition, loss, and regain in females with classic Rett syndrome
Source: J Neurodev Disord. 2026 Mar 12;18:20. doi: 10.1186/s11689-026-09680-6 (PMC13094048; doi:10.1186/s11689-026-09680-6)
Supplement: Supplementary file 3 — Supplementary Material 3 [file 11689_2026_9680_MOESM3_ESM.pdf]

Table S3: Developmental skill mapping between protocols 5201 and 5211

	Skill Name Utilized for Manuscript	5211 Skill Name	5201 Skill Name
Gross Motor	Lift Head	Lift head while lying down	*Not captured*
	Roll From Tummy	Rolled from tummy to back	Roll front to back
	Not included	*Not captured*	Roll back to front
	Sit With Support	Sat with support when placed	*Not captured*
	Sit Without Support	Sat without support when placed	Sits when placed
	Come to Sit	Come to sitting	Come to sit
	Crawl	Crawled	Crawl
	Stand With Support	Stood while holding on	*Not captured*
	Pull To Stand	Pulled to standing	Pull to stand
	Walk With Support	Cruised around furniture or holding on to someone	Walk with support
	Stand Independently	Stood independently	*Not included*
	Walk Independently	Walked independently	Walk independently
	Run 10 Feet	Ran 10 feet wthout falling	*Not included*
	Up Stairs With Help	Climbed up stairs with help	*Not included*
	Up Stairs Without Help	Climbed up stairs without help	Stairs up wihtout help
	Down Stairs With Help	Climbed down stairs with help	*Not included*
	Down Stairs Without Help	Climbed down stairs without help	Stairs down without help
	Not included	*Not captured*	Pedal tricycle
Fine Motor	Hold Bottle	Held bottle or cup un-propped	Holds bottle
	Reach for Toy	Reached for toy	Reach for toy
	Raking Grasp	Used raking grasp to retrieve and object	*Not captured*
	Transfer Objects	Transferred an object fromone hand to the other	Transfer
	Pincer Grasp	Used a pincer grasp	Pincer grasp
	Finger Feeding	Finger feed	Finger feed
	Turn Pages in Book	Turned pages in book	*Not captured*
Expressive	Social Smile	Social smile	Social smile
	Cooing	Cooed	Coo
	Babbling	Babbled	Babble
	Words With Meaning	Used words with meaning	Single words with meaning
	Spoken Phrases	Spoken in phrases	Phrases
	Wave Bye	Waved bye=bye	Gestures (waves)
	Point for Wants	Pointed for something they want	Points for wants
	Shared Stories	Shared stories	*Not captured*
Receptive	Quiet To Voice	Quieted or been soothed by the soundof a familiar adult's voice	Quiet to voice
	Responds To Sounds	Responded to sounds	*Not captured*
	Play Peek-A-Boo	Played Peekaboo	*Not captured*
	Respond To Familiar Words	Responded to familiar names/words such as mama or doggy	*Not captured*
	Respond To Own Name	Responded to own name	*Not captured*
	Inhibit To No	Inhibited to "no" or respnded to different tones of voice	Inhibit to "No"
	Follows Commands With Gesture	Followed a command with a gesture	Follow command with gesture
	Follows Commands Without Gesture	Followed a command without a gesture	Follow command without gesture
	Identify Body Parts	Identified body parts (pointed with eyes or fingers)	*Not captured*
	Point to 1 Color	Pointed to 1 color when asked (with fingers or eyes)	*Not captured*
Social/Adaptive	Like Being Held	Like being held	Like being held
	Attention to Loud Sound	Paid attention to loud or prolonged sound	Stopped being aurally attentive
	Not included	*Not captured*	Stopped being visually attentive
	Eyes Fix and Follow	Fixed and followed an object with eyes	Fix and follow
	Play Pat-A-Cake	Played pat-a-cake	*Not captured*
	Desire Social Attention	Desired social attention	*Not captured*
	Imitate Peers	Imitated peers in play activity	*Not captured*
	Been Independent	Been Independent - wanted to do things by themselves	*Not captured*
	Drink From Cup Without Help	Taken a drink from a cup held without assistance	*Not captured*
	Uses Utensils With Help	Used a spoon/fork to eat with assistance	*Not captured*
	Uses Utensils Without Help	Used a spoon/fork to eat without assistance	*Not captured*
