# Supplementary material for: Trajectory of skill acquisition, loss, and regain in females with classic Rett syndrome
Source: J Neurodev Disord. 2026 Mar 12;18:20. doi: 10.1186/s11689-026-09680-6 (PMC13094048; doi:10.1186/s11689-026-09680-6)
Supplement: Supplementary file 5 — Supplementary Material 5 [file 11689_2026_9680_MOESM5_ESM.pdf]

Figure S2: Cumulative incidence curves of skill loss. Censored data points are shown as cross lines. The red vertical line is the median age of loss for that specific skill.

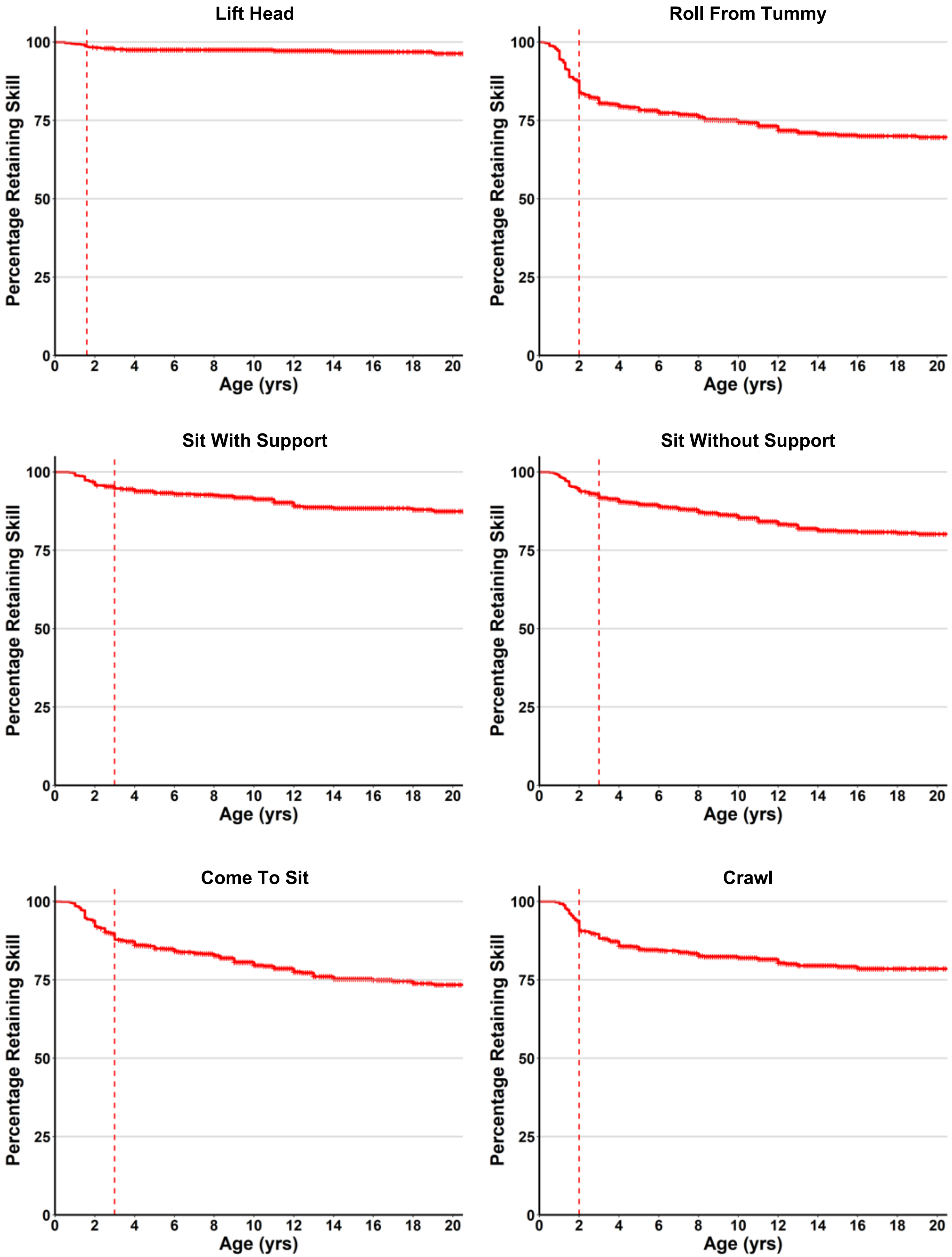

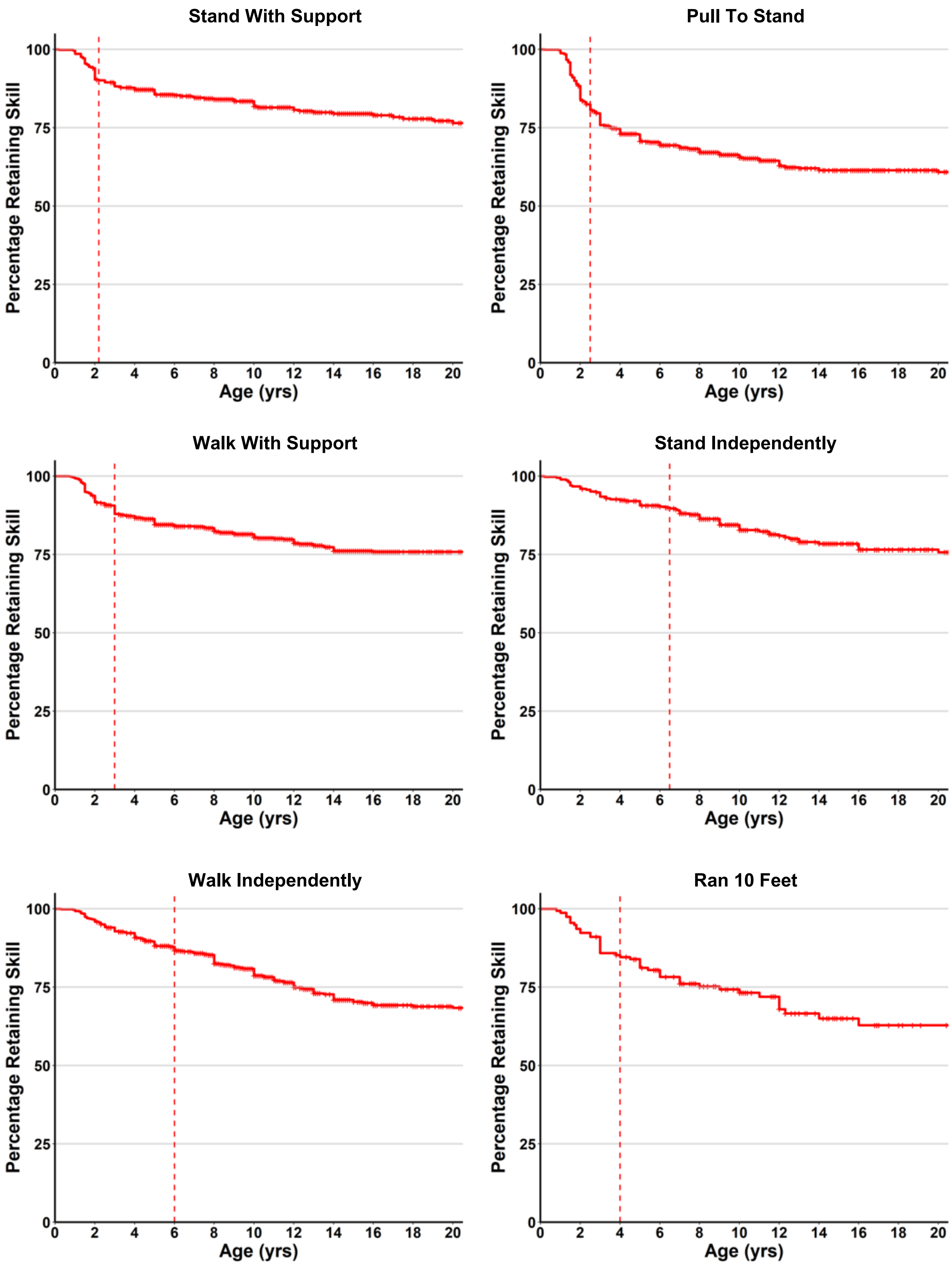
Stand With Support
Pull To Stand
Walk With Support
Stand Independently
Walk Independently
Ran 10 Feet
Percentage Retaining Skill
Age (yrs)
0
25
50
75
100
0 2 4 6 8 10 12 14 16 18 20

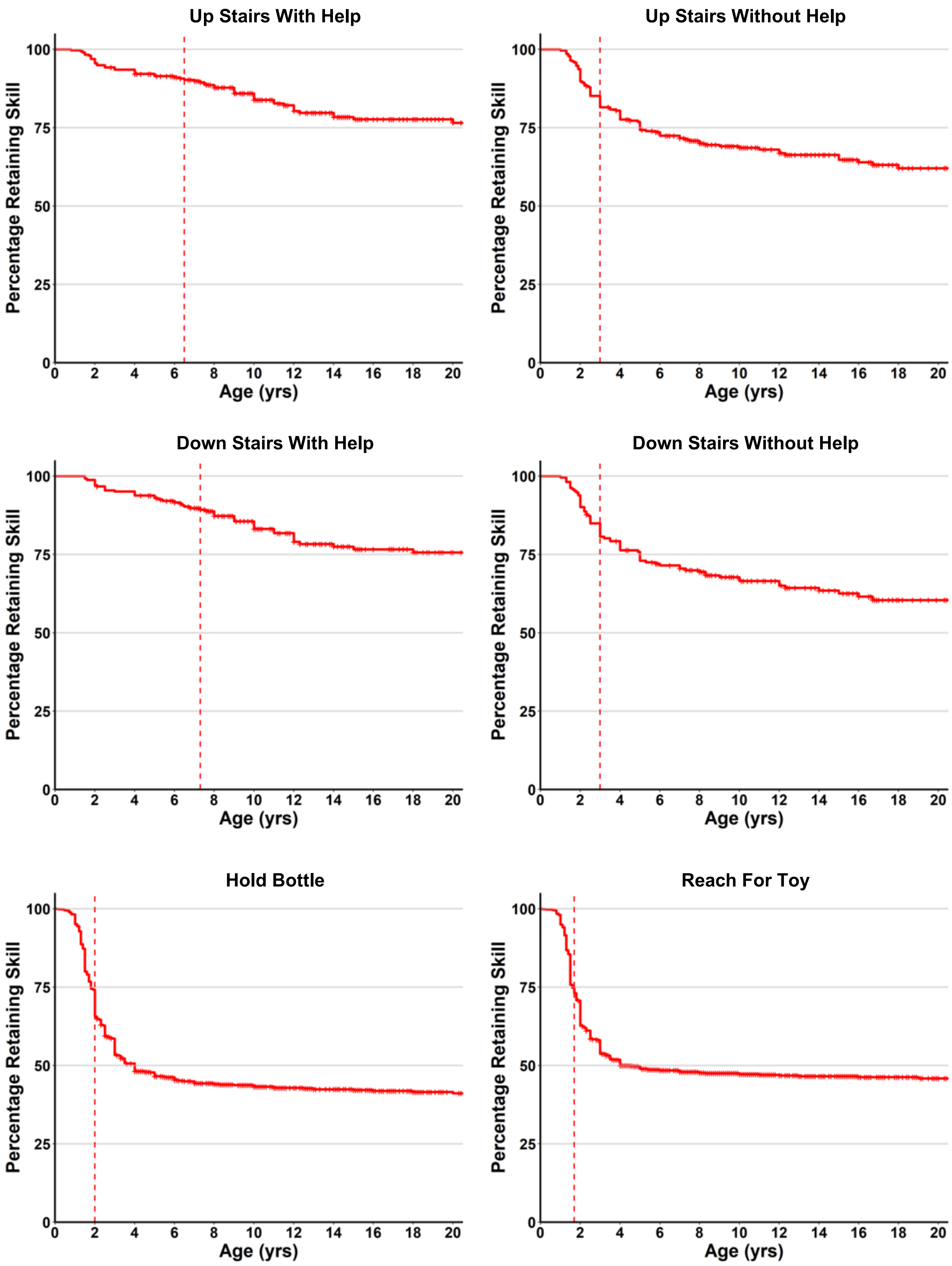

Up Stairs With Help
Up Stairs Without Help
Down Stairs With Help
Down Stairs Without Help
Hold Bottle
Reach For Toy
Percentage Retaining Skill
Age (yrs)
100
75
50
25
0
0 2 4 6 8 10 12 14 16 18 20

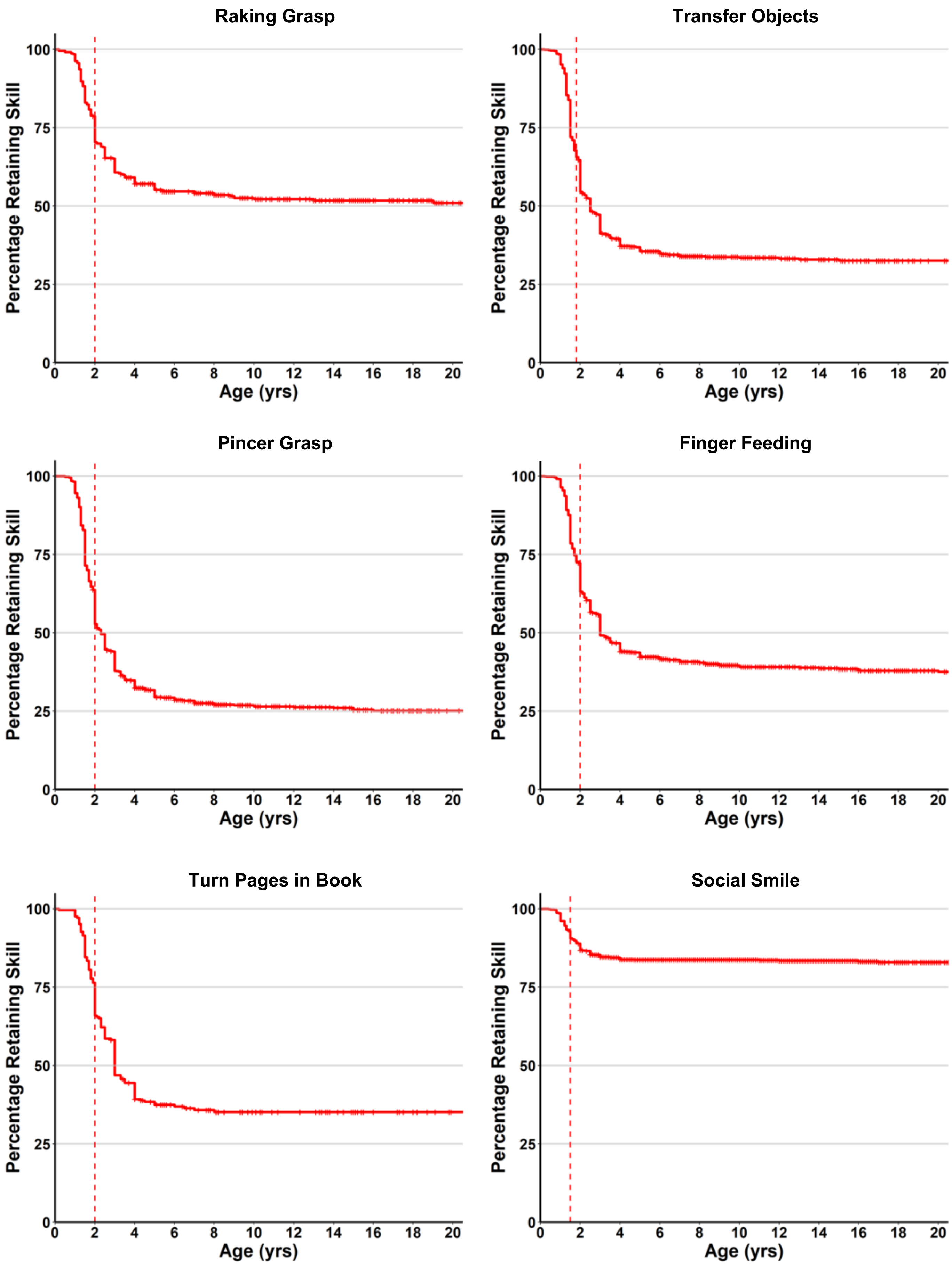

Raking Grasp
Transfer Objects
Pincer Grasp
Finger Feeding
Turn Pages in Book
Social Smile
Percentage Retaining Skill
Age (yrs)
0
25
50
75
100
0
2
4
6
8
10
12
14
16
18
20

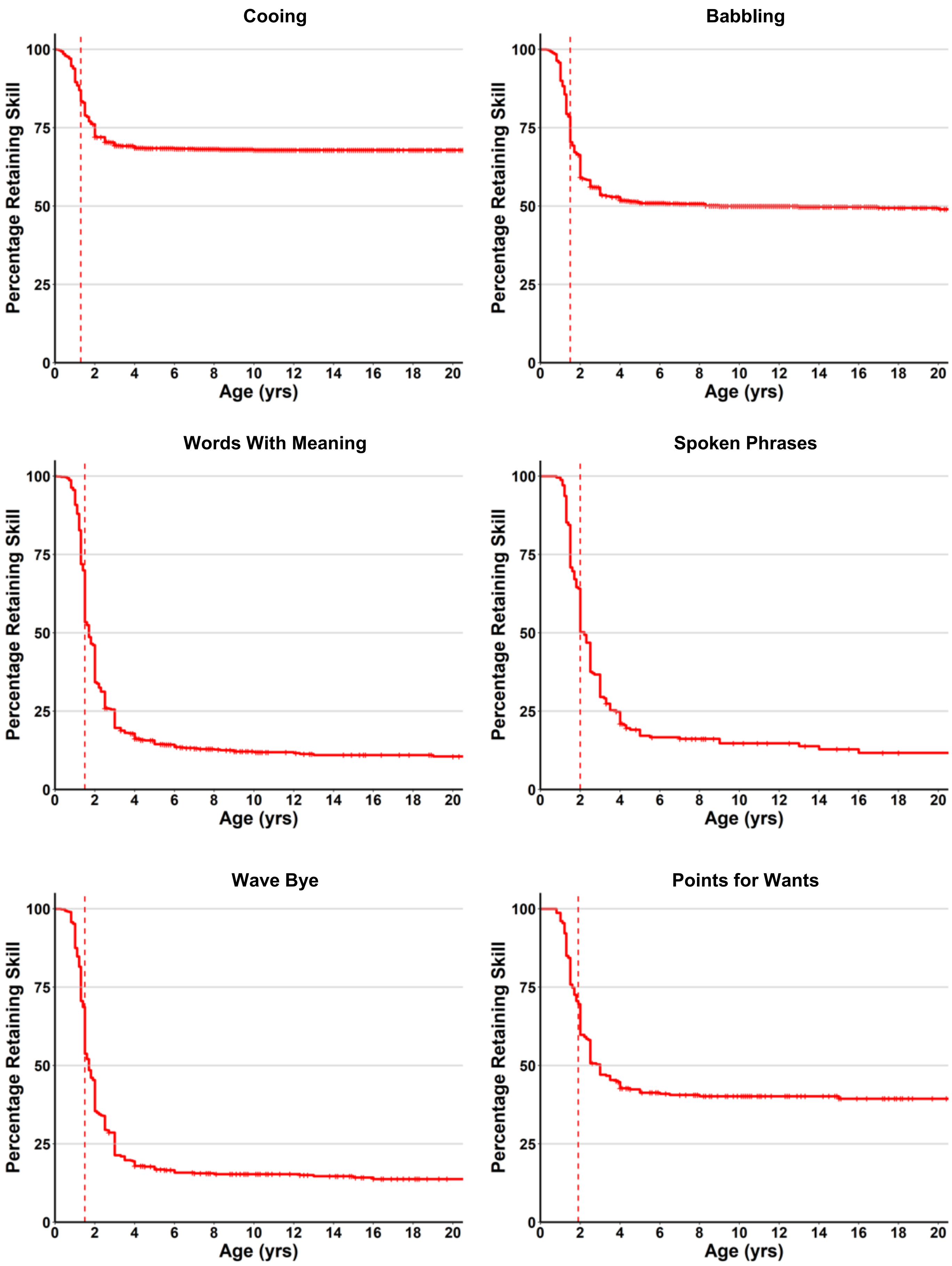
Cooing
Babbling
Words With Meaning
Spoken Phrases
Wave Bye
Points for Wants
Percentage Retaining Skill
Age (yrs)
100
75
50
25
0
0
2
4
6
8
10
12
14
16
18
20

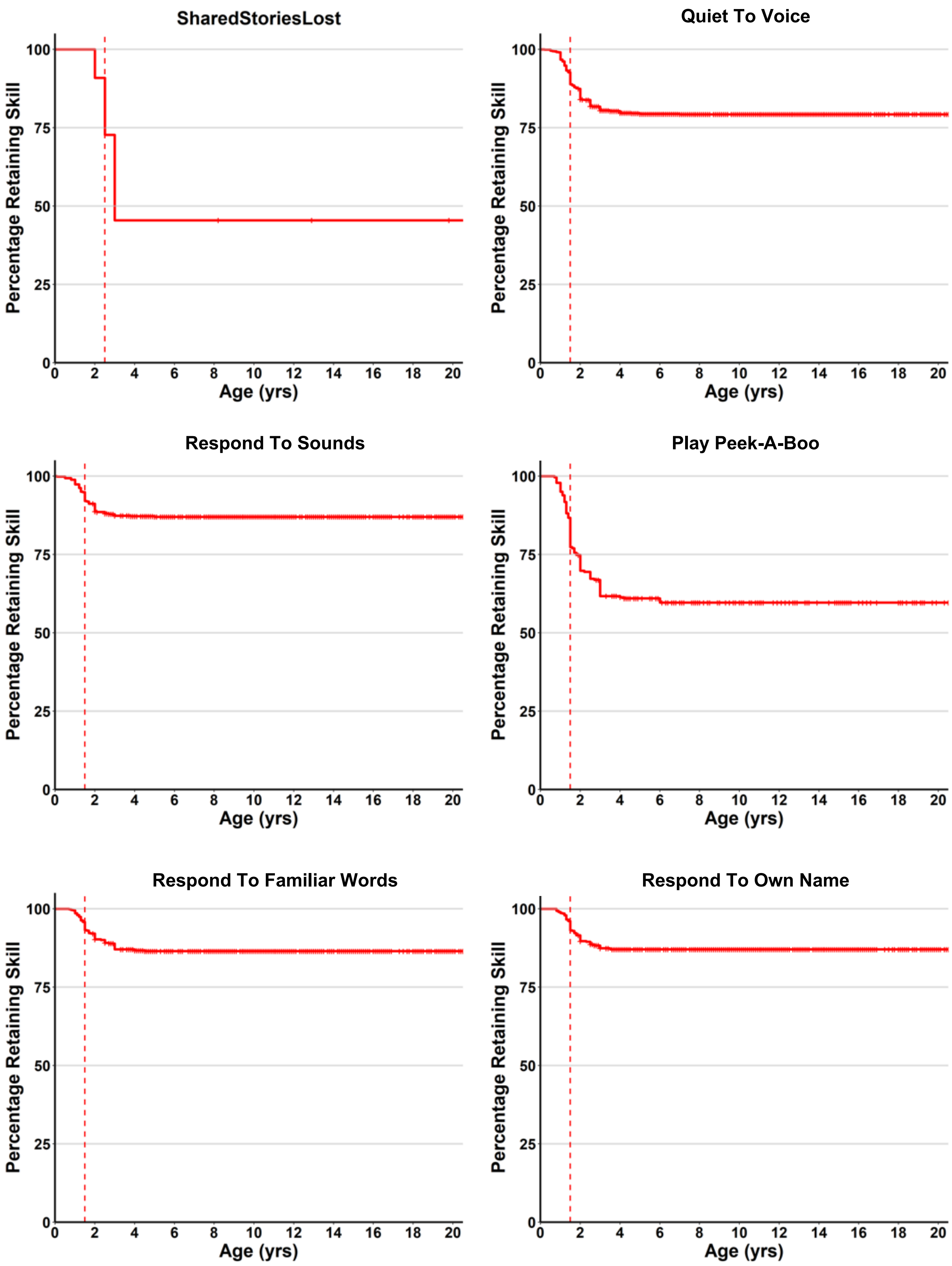

SharedStoriesLost
Quiet To Voice
Respond To Sounds
Play Peek-A-Boo
Respond To Familiar Words
Respond To Own Name
Percentage Retaining Skill
Age (yrs)
100
75
50
25
0
0 2 4 6 8 10 12 14 16 18 20

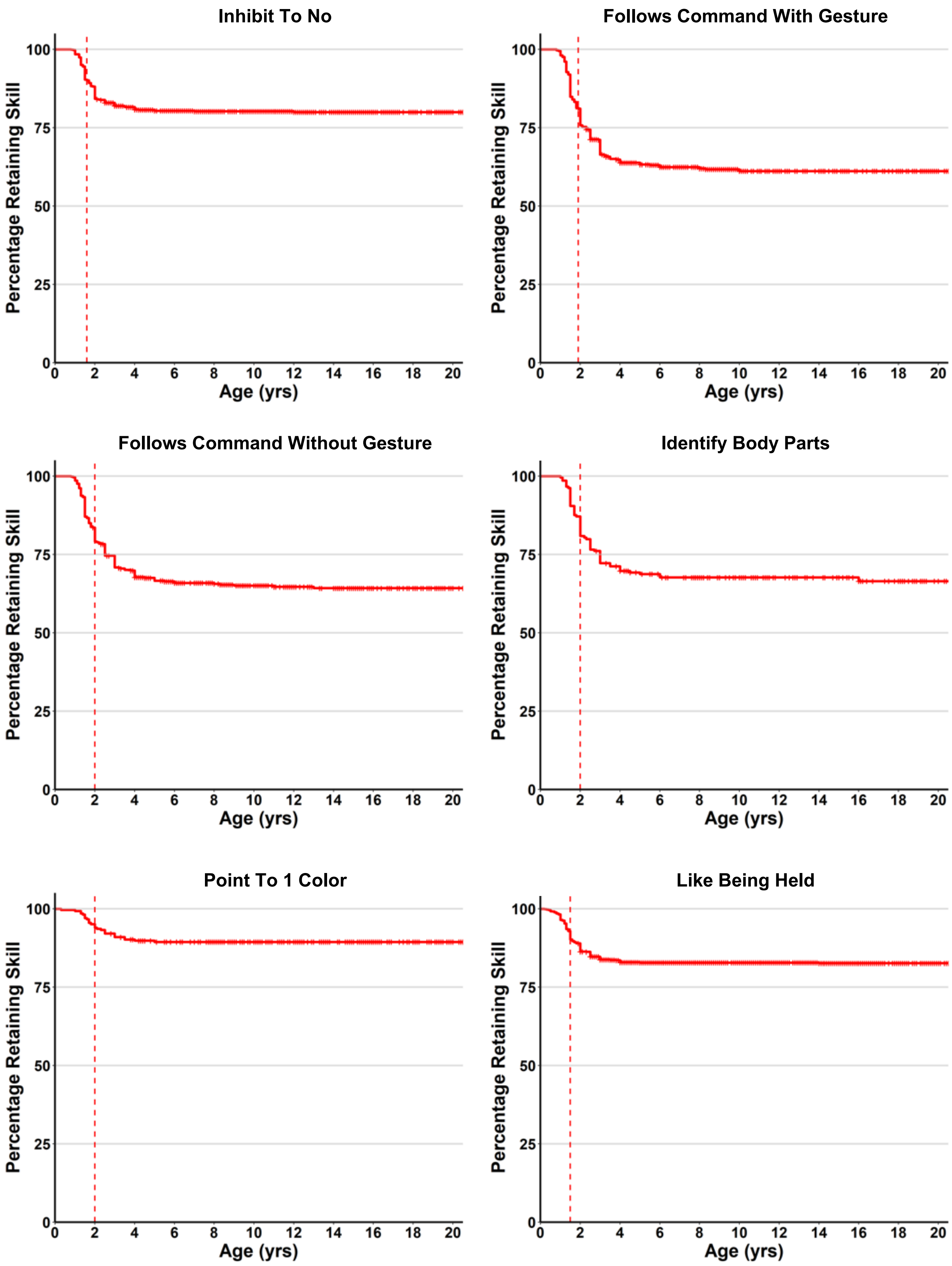

Inhibit To No
Follows Command With Gesture
Follows Command Without Gesture
Identify Body Parts
Point To 1 Color
Like Being Held
Percentage Retaining Skill
Age (yrs)
0
25
50
75
100
0
2
4
6
8
10
12
14
16
18
20

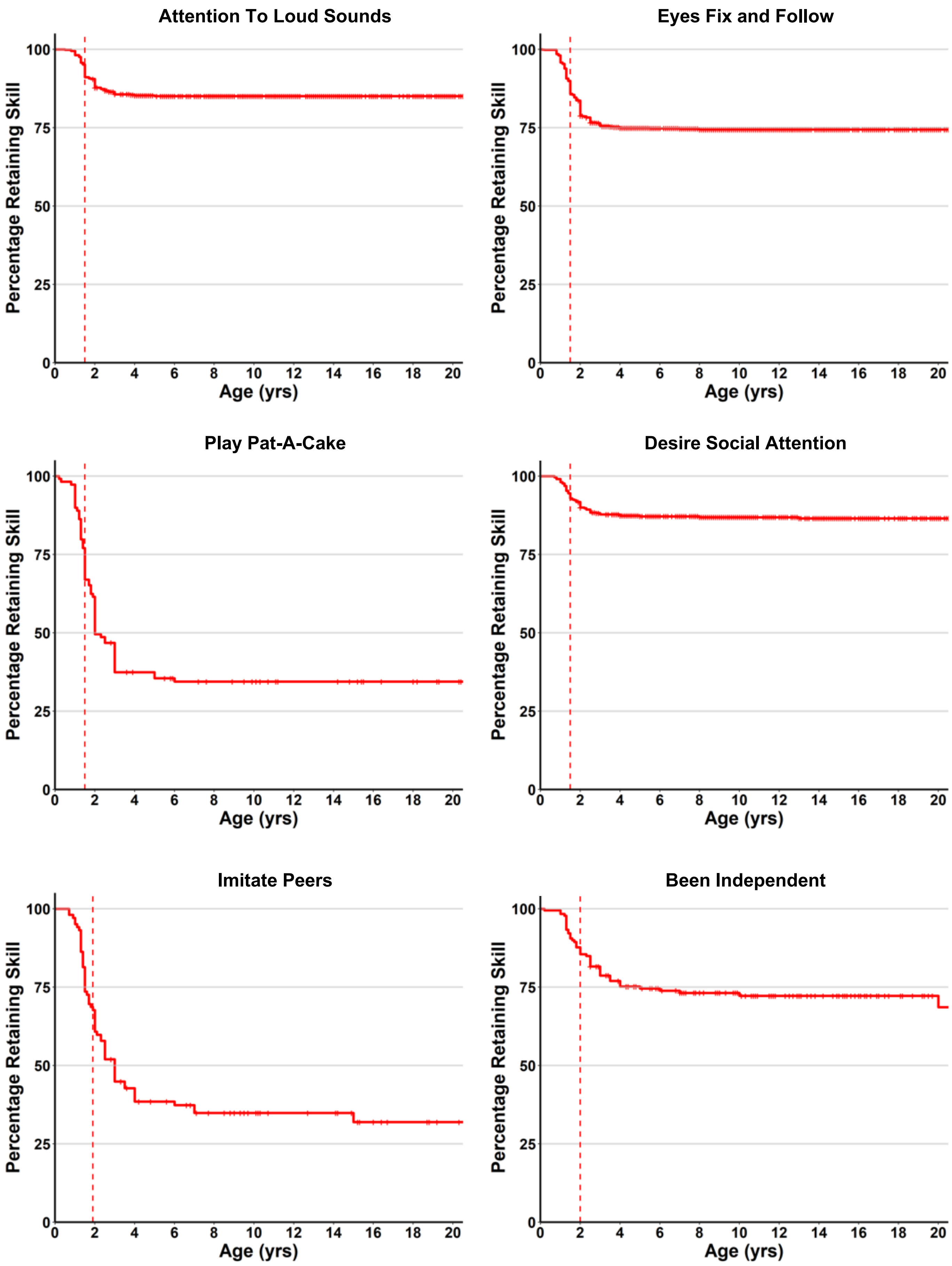
Attention To Loud Sounds
Eyes Fix and Follow
Play Pat-A-Cake
Desire Social Attention
Imitate Peers
Been Independent
Percentage Retaining Skill
Age (yrs)
0
25
50
75
100
0
2
4
6
8
10
12
14
16
18
20

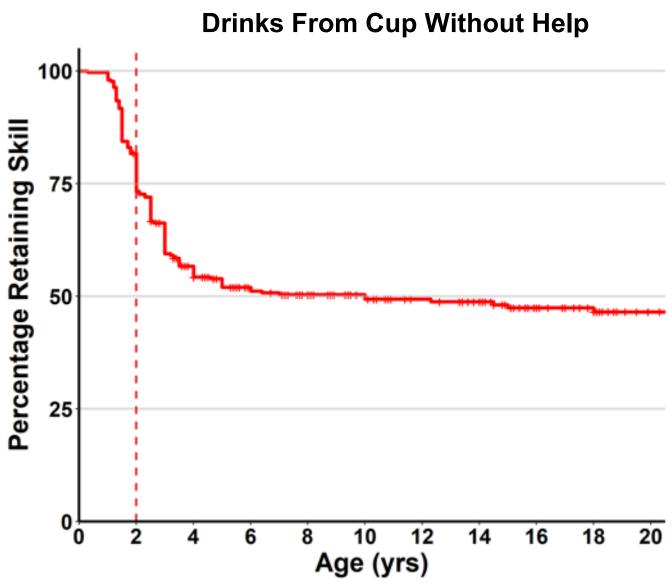

Drinks From Cup Without Help
Percentage Retaining Skill
100
75
50
25
0
0
2
4
6
8
10
12
14
16
18
20
Age (yrs)

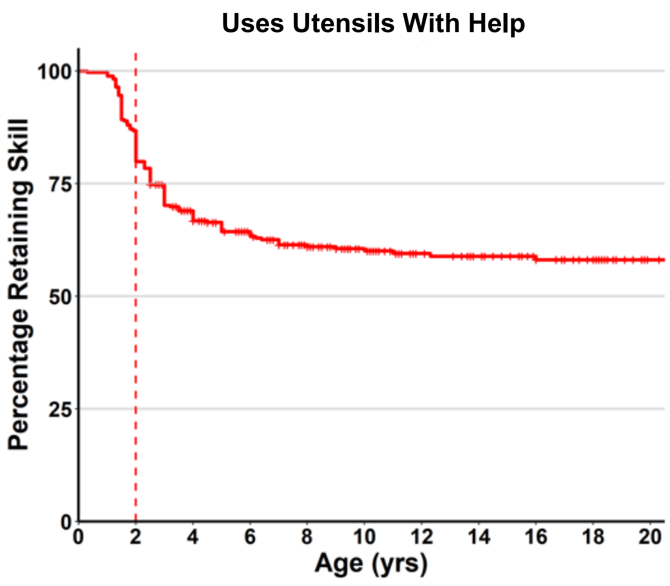

Uses Utensils With Help
Percentage Retaining Skill
100
75
50
25
0
0
2
4
6
8
10
12
14
16
18
20
Age (yrs)

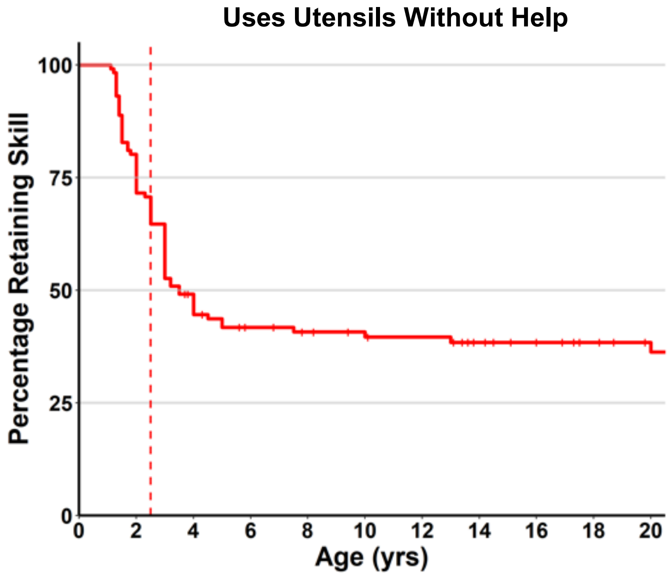

Uses Utensils Without Help
Percentage Retaining Skill
100
75
50
25
0
0
2
4
6
8
10
12
14
16
18
20
Age (yrs)
