# Supplementary material for: Trajectory of skill acquisition, loss, and regain in females with classic Rett syndrome
Source: J Neurodev Disord. 2026 Mar 12;18:20. doi: 10.1186/s11689-026-09680-6 (PMC13094048; doi:10.1186/s11689-026-09680-6)
Supplement: Supplementary file 6 — Supplementary Material 6 [file 11689_2026_9680_MOESM6_ESM.pdf]

Figure S3: Cumulative incidence curves of time from skill gain to loss. Censored data points are shown as cross lines. The red vertical line is the median time of gain to loss for that specific skill.

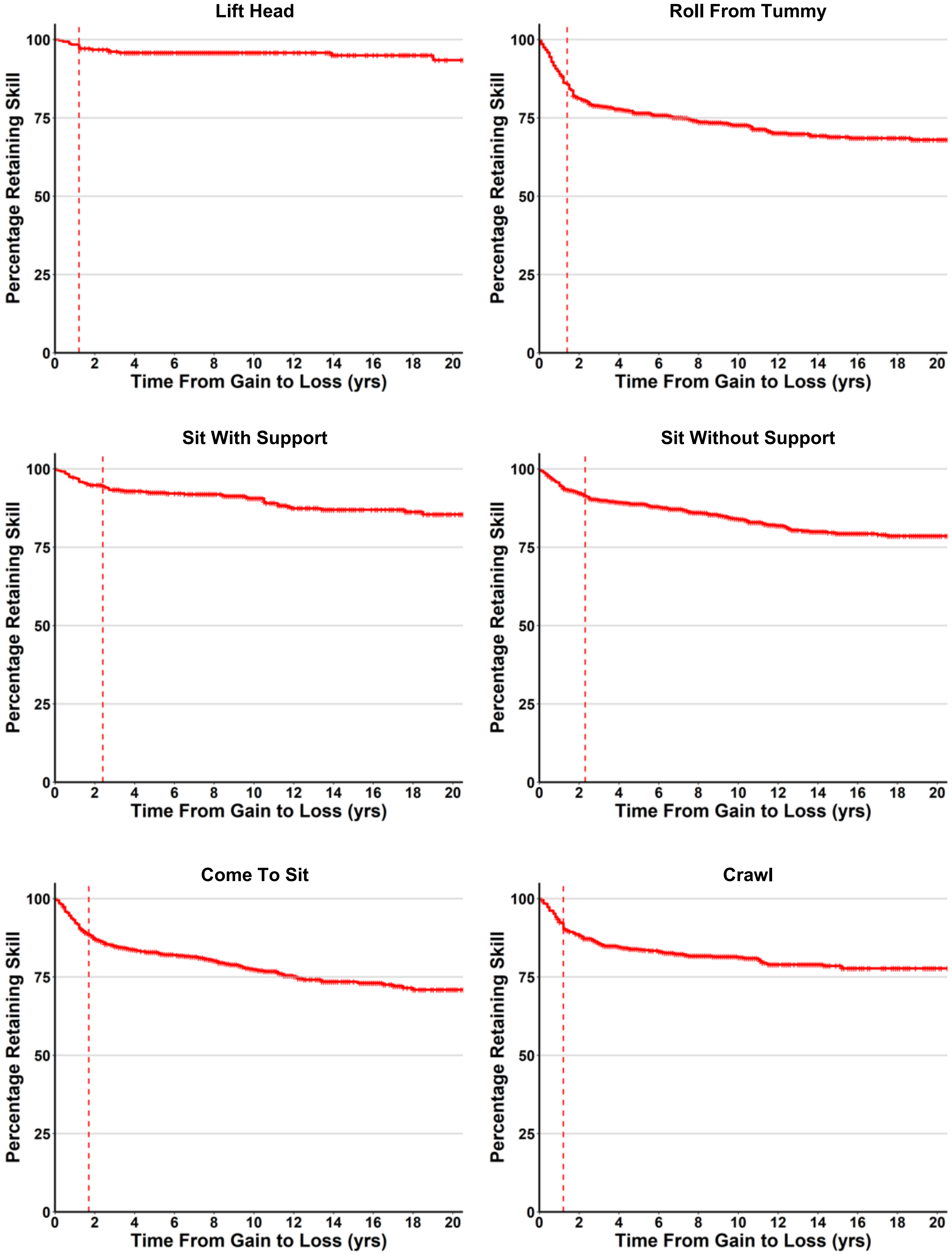

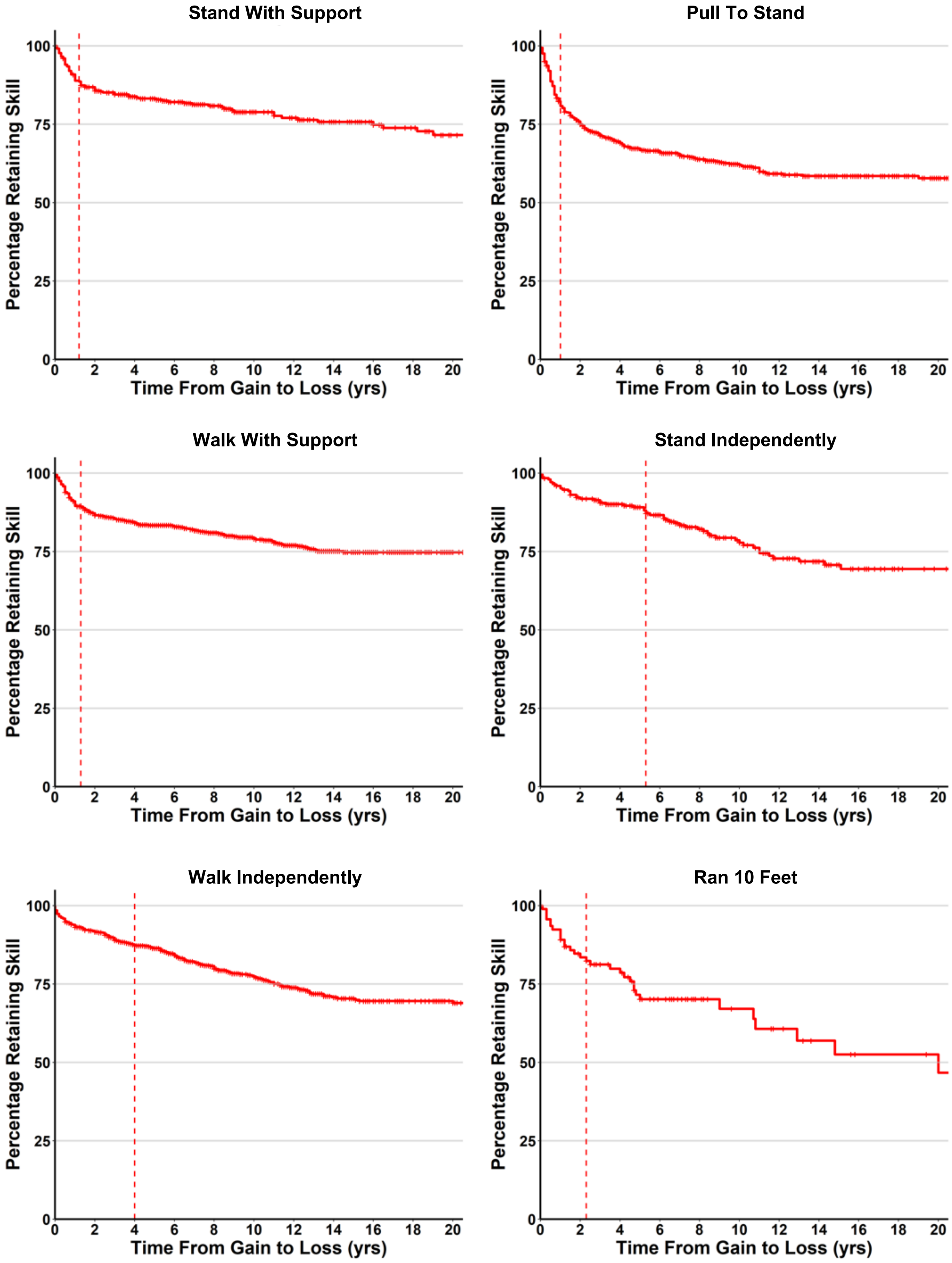
Stand With Support
Pull To Stand
Walk With Support
Stand Independently
Walk Independently
Ran 10 Feet
Percentage Retaining Skill
Time From Gain to Loss (yrs)
0
25
50
75
100
0
2
4
6
8
10
12
14
16
18
20

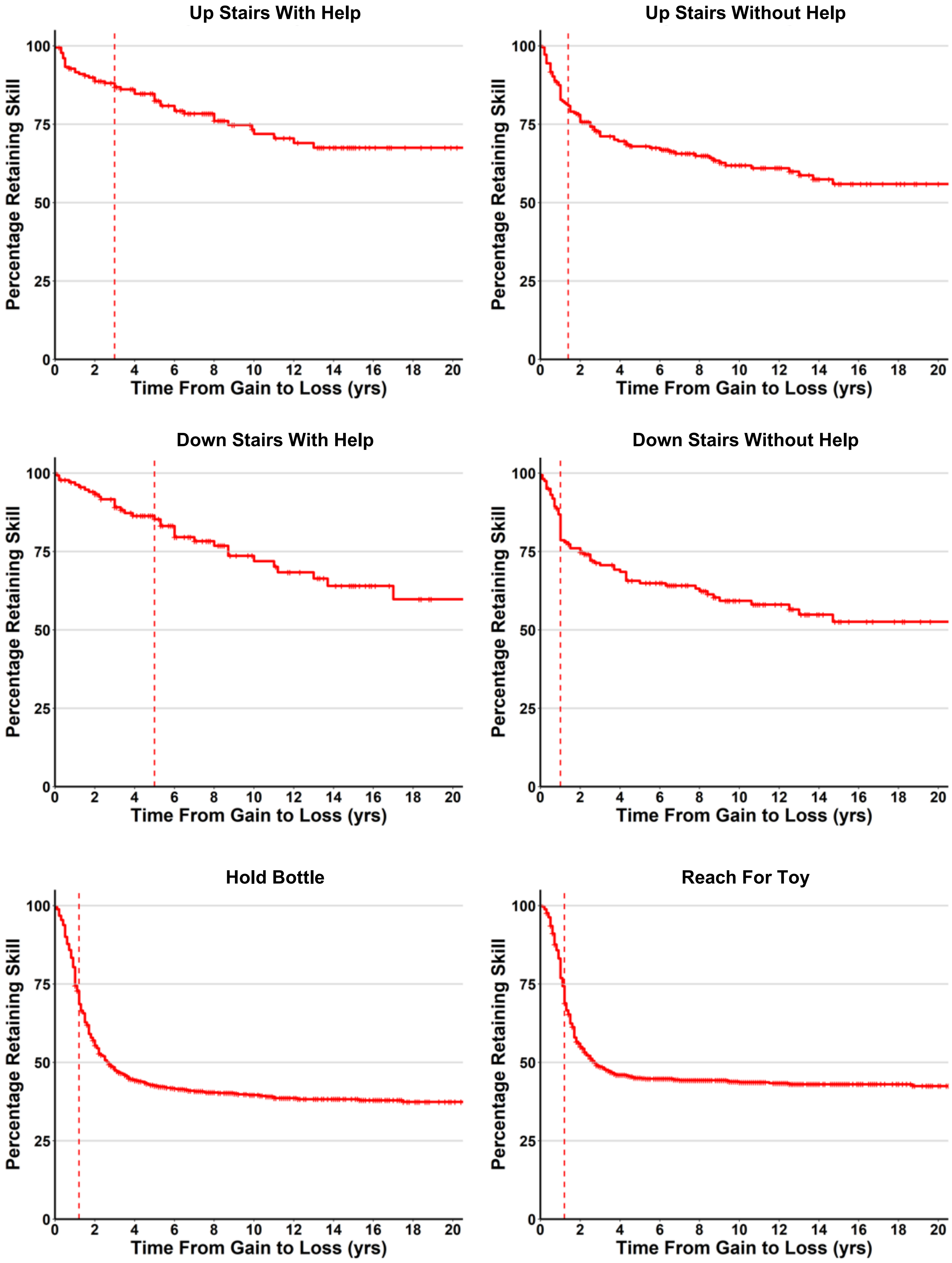

Up Stairs With Help
Up Stairs Without Help
Down Stairs With Help
Down Stairs Without Help
Hold Bottle
Reach For Toy
Percentage Retaining Skill
Time From Gain to Loss (yrs)
0
25
50
75
100
0
2
4
6
8
10
12
14
16
18
20

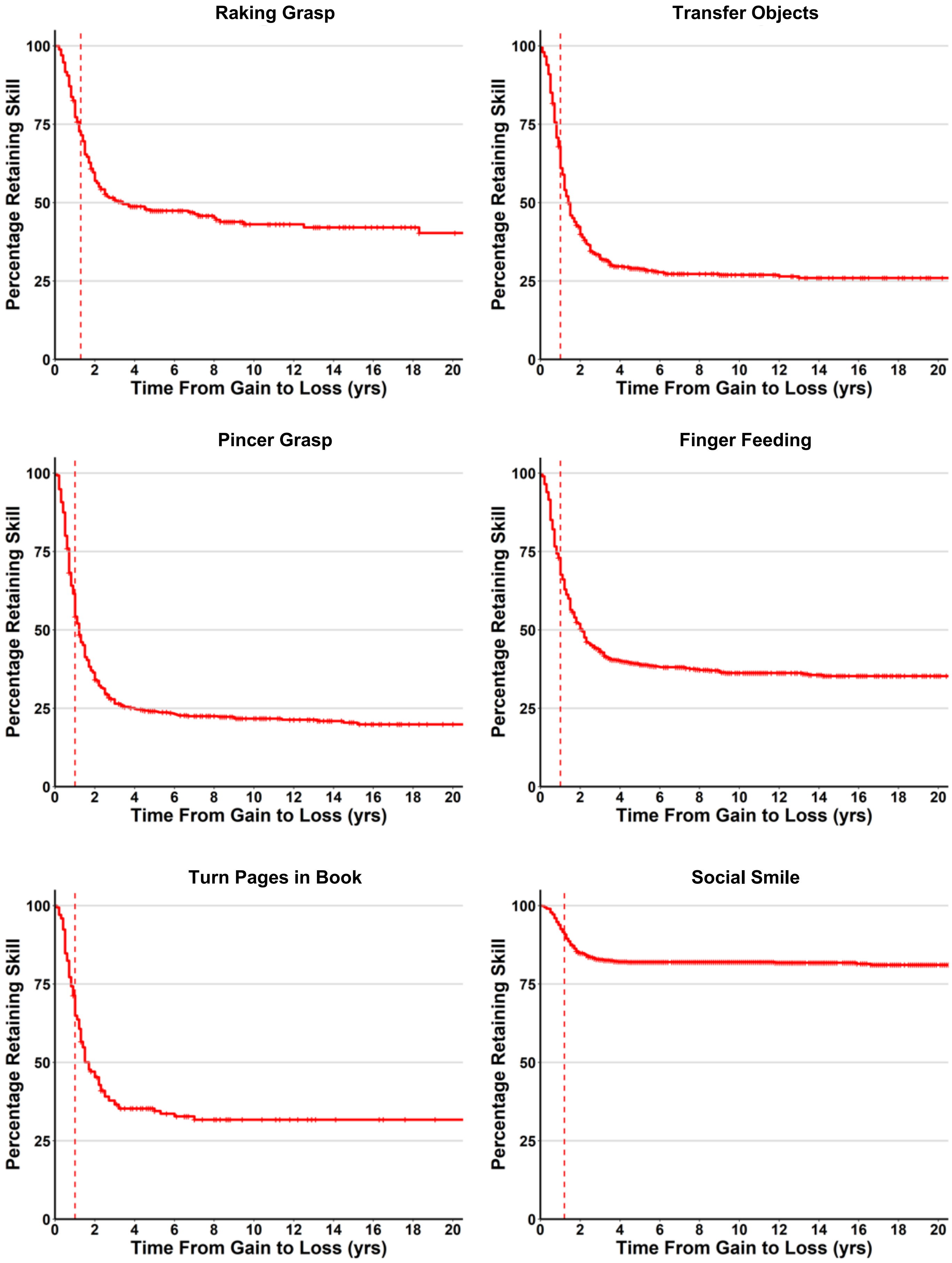

Raking Grasp
Percentage Retaining Skill
0
25
50
75
100
0 2 4 6 8 10 12 14 16 18 20
Time From Gain to Loss (yrs)
Transfer Objects
Percentage Retaining Skill
0
25
50
75
100
0 2 4 6 8 10 12 14 16 18 20
Time From Gain to Loss (yrs)
Pincer Grasp
Percentage Retaining Skill
0
25
50
75
100
0 2 4 6 8 10 12 14 16 18 20
Time From Gain to Loss (yrs)
Finger Feeding
Percentage Retaining Skill
0
25
50
75
100
0 2 4 6 8 10 12 14 16 18 20
Time From Gain to Loss (yrs)
Turn Pages in Book
Percentage Retaining Skill
0
25
50
75
100
0 2 4 6 8 10 12 14 16 18 20
Time From Gain to Loss (yrs)
Social Smile
Percentage Retaining Skill
0
25
50
75
100
0 2 4 6 8 10 12 14 16 18 20
Time From Gain to Loss (yrs)

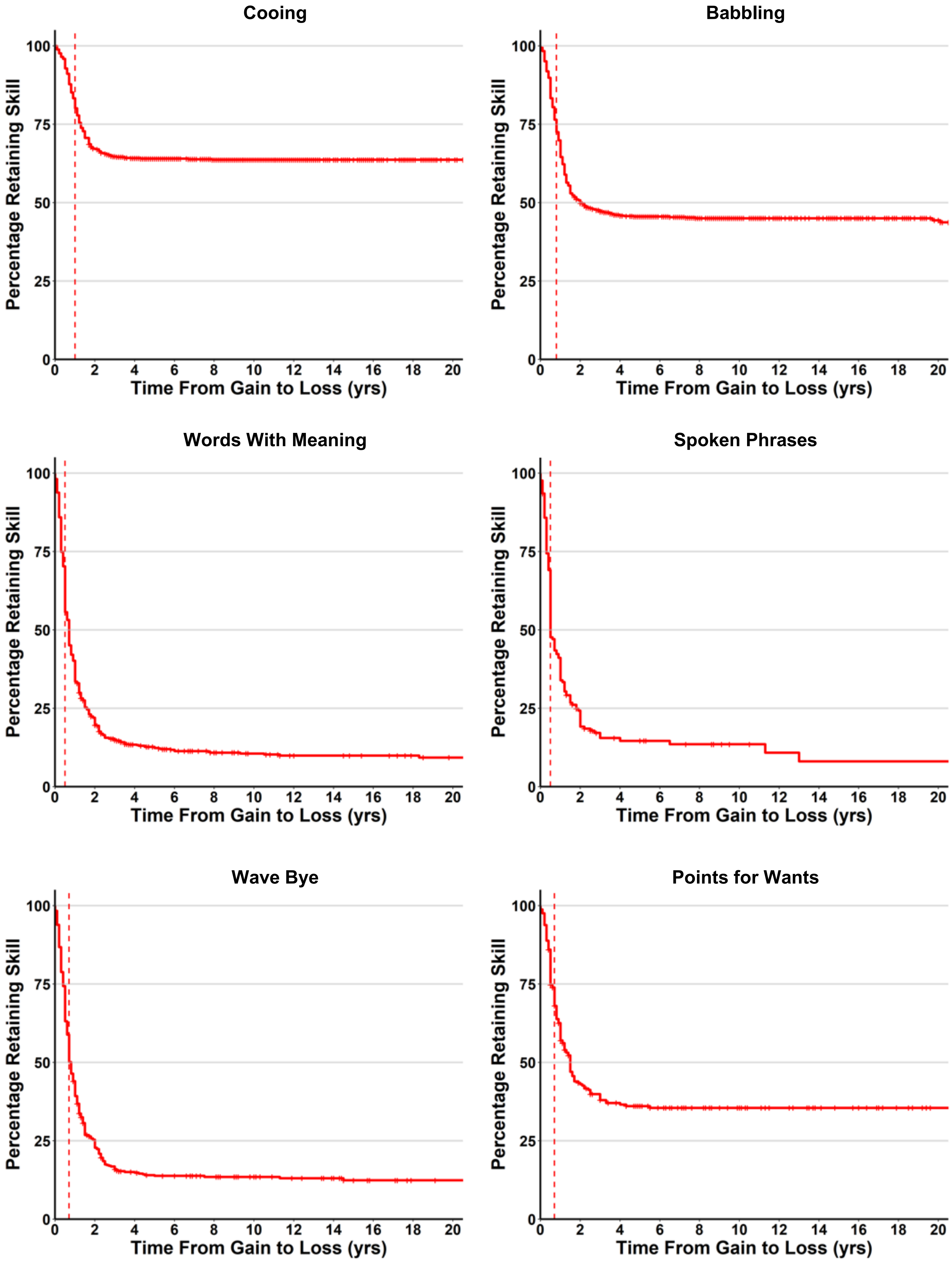
Cooing
Babbling
Words With Meaning
Spoken Phrases
Wave Bye
Points for Wants
Percentage Retaining Skill
Time From Gain to Loss (yrs)
0
25
50
75
100
0
2
4
6
8
10
12
14
16
18
20

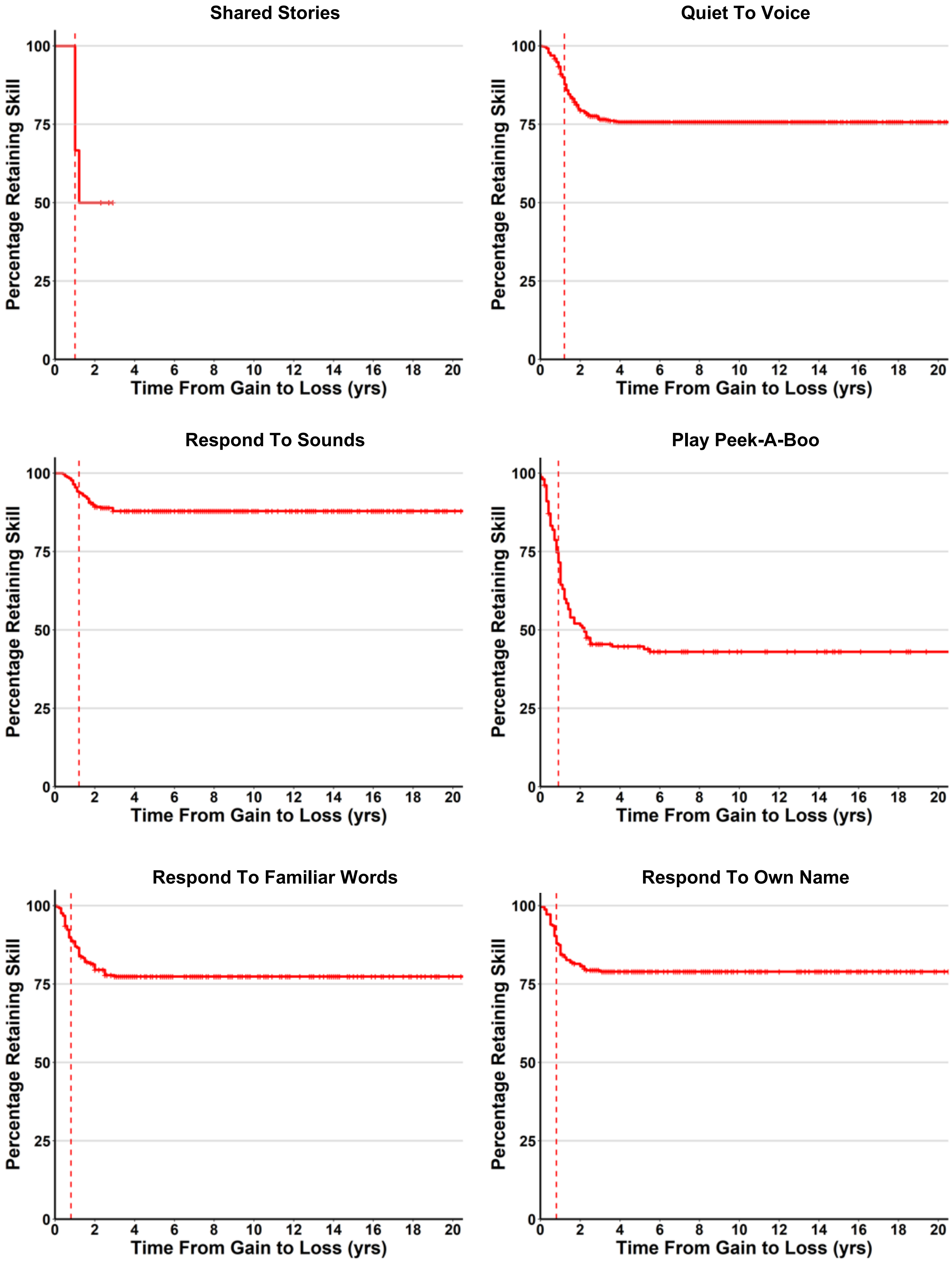
Shared Stories
Quiet To Voice
Respond To Sounds
Play Peek-A-Boo
Respond To Familiar Words
Respond To Own Name
Percentage Retaining Skill
Time From Gain to Loss (yrs)
0
25
50
75
100
0
2
4
6
8
10
12
14
16
18
20

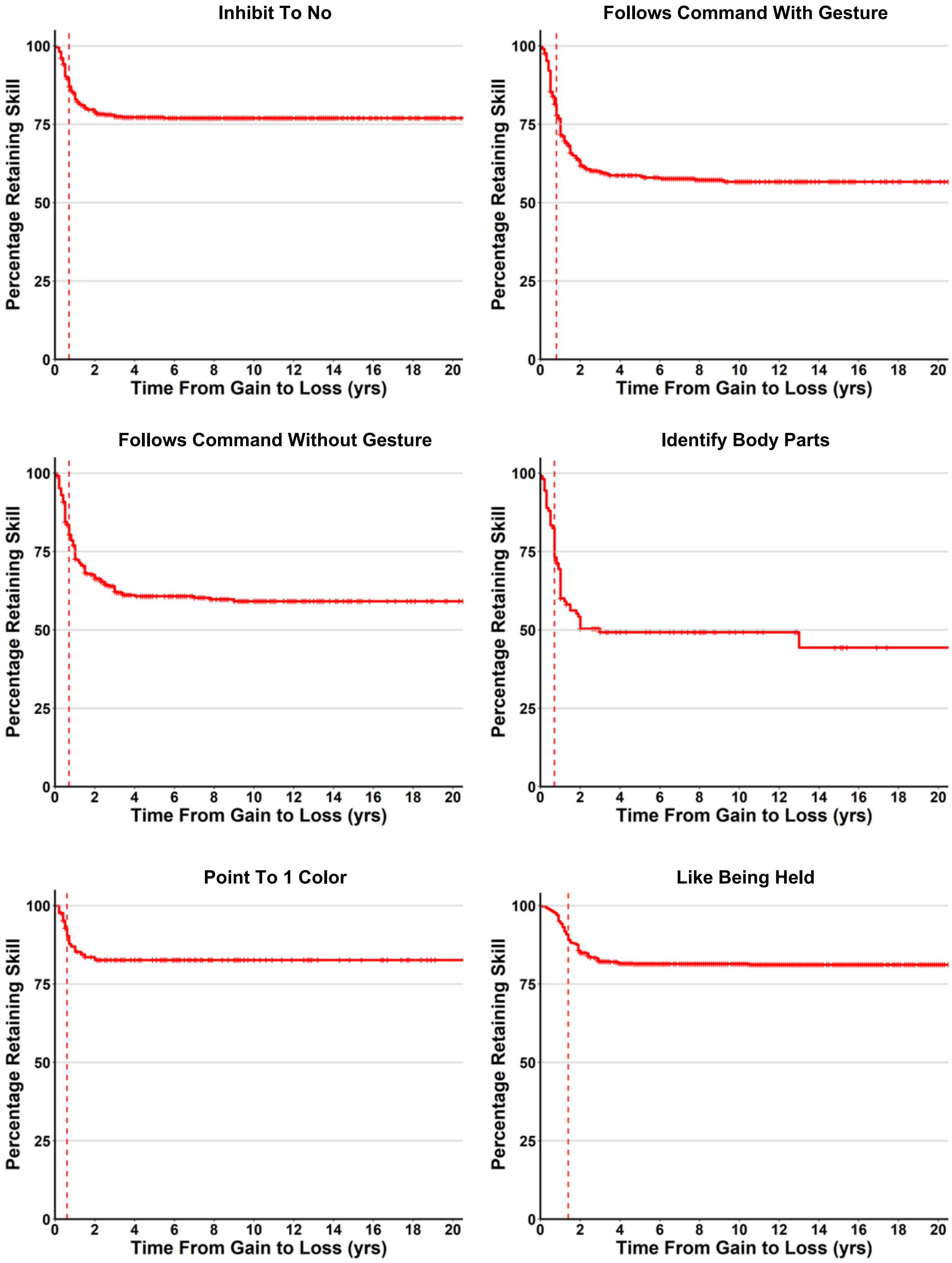
Inhibit To No
Follows Command With Gesture
Follows Command Without Gesture
Identify Body Parts
Point To 1 Color
Like Being Held
Percentage Retaining Skill
0
25
50
75
100
Time From Gain to Loss (yrs)
0
2
4
6
8
10
12
14
16
18
20

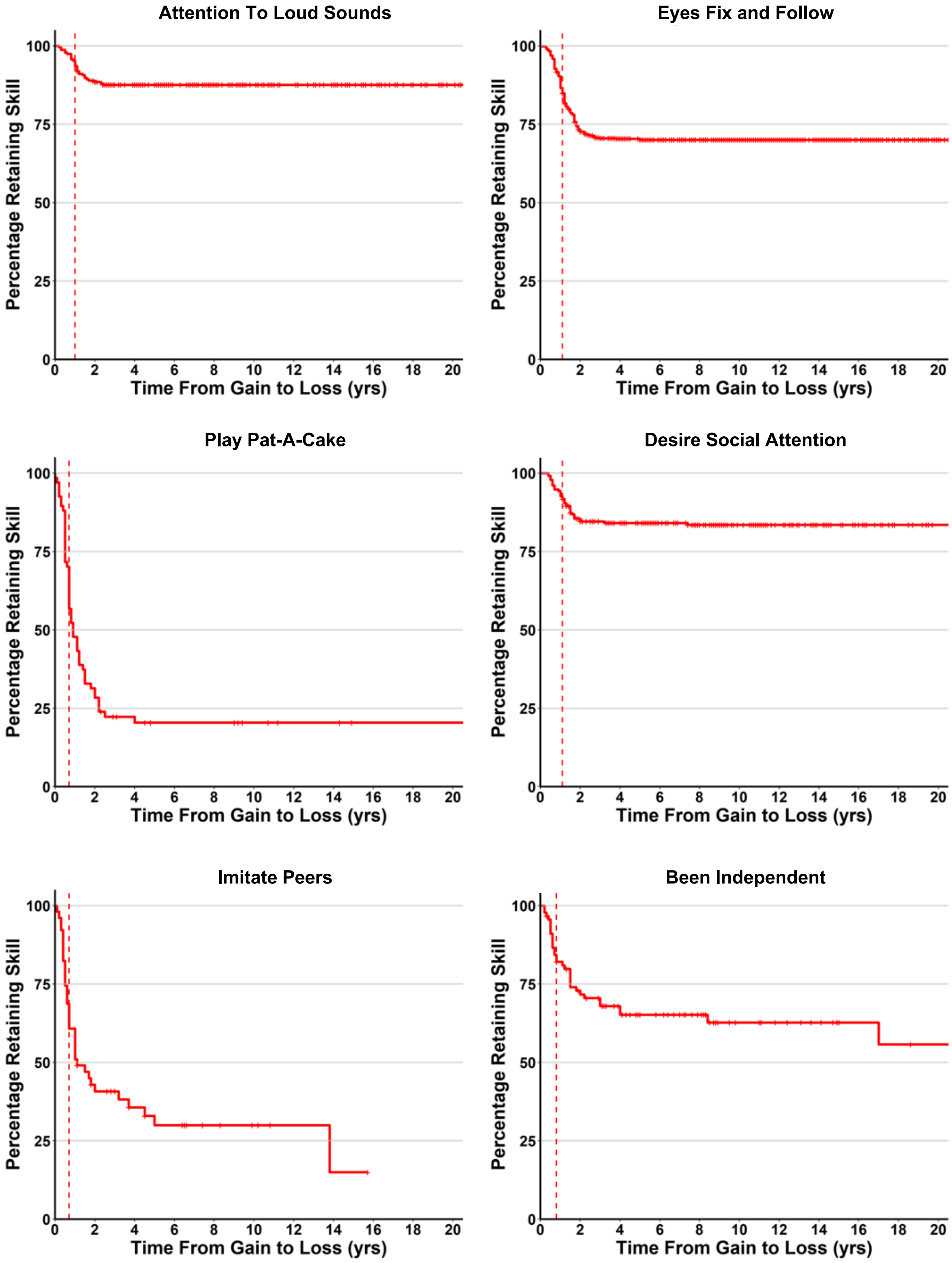

Attention To Loud Sounds
Eyes Fix and Follow
Play Pat-A-Cake
Desire Social Attention
Imitate Peers
Been Independent
Percentage Retaining Skill
Time From Gain to Loss (yrs)
0
25
50
75
100
0
2
4
6
8
10
12
14
16
18
20

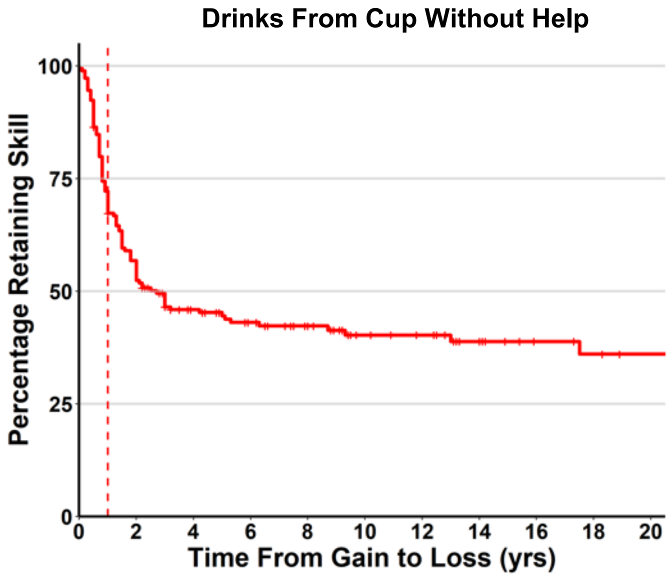
Drinks From Cup Without Help
Percentage Retaining Skill
100
75
50
25
0
0
2
4
6
8
10
12
14
16
18
20
Time From Gain to Loss (yrs)

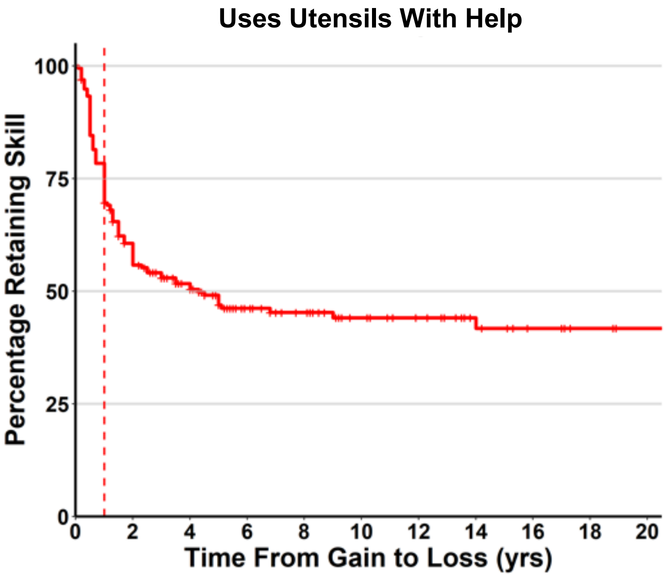
Uses Utensils With Help
Percentage Retaining Skill
100
75
50
25
0
0
2
4
6
8
10
12
14
16
18
20
Time From Gain to Loss (yrs)

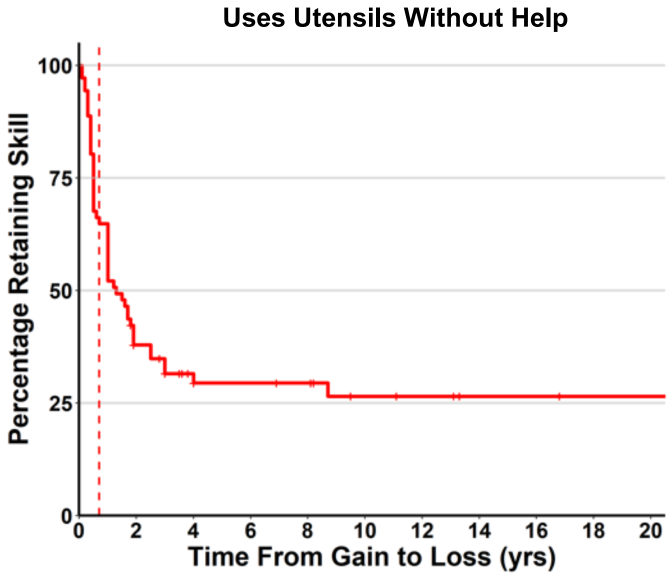
Uses Utensils Without Help
Percentage Retaining Skill
100
75
50
25
0
0
2
4
6
8
10
12
14
16
18
20
Time From Gain to Loss (yrs)
