# Supplementary material for: Trajectory of skill acquisition, loss, and regain in females with classic Rett syndrome
Source: J Neurodev Disord. 2026 Mar 12;18:20. doi: 10.1186/s11689-026-09680-6 (PMC13094048; doi:10.1186/s11689-026-09680-6)
Supplement: Supplementary file 7 — Supplementary Material 7 [file 11689_2026_9680_MOESM7_ESM.pdf]

**Figure S4: Cumulative incidence curves of age of skill regain.** Censored data points are shown as cross lines. The red vertical line is the median age of regain for that specific skill; the black dashed line demarks 6 years of age.

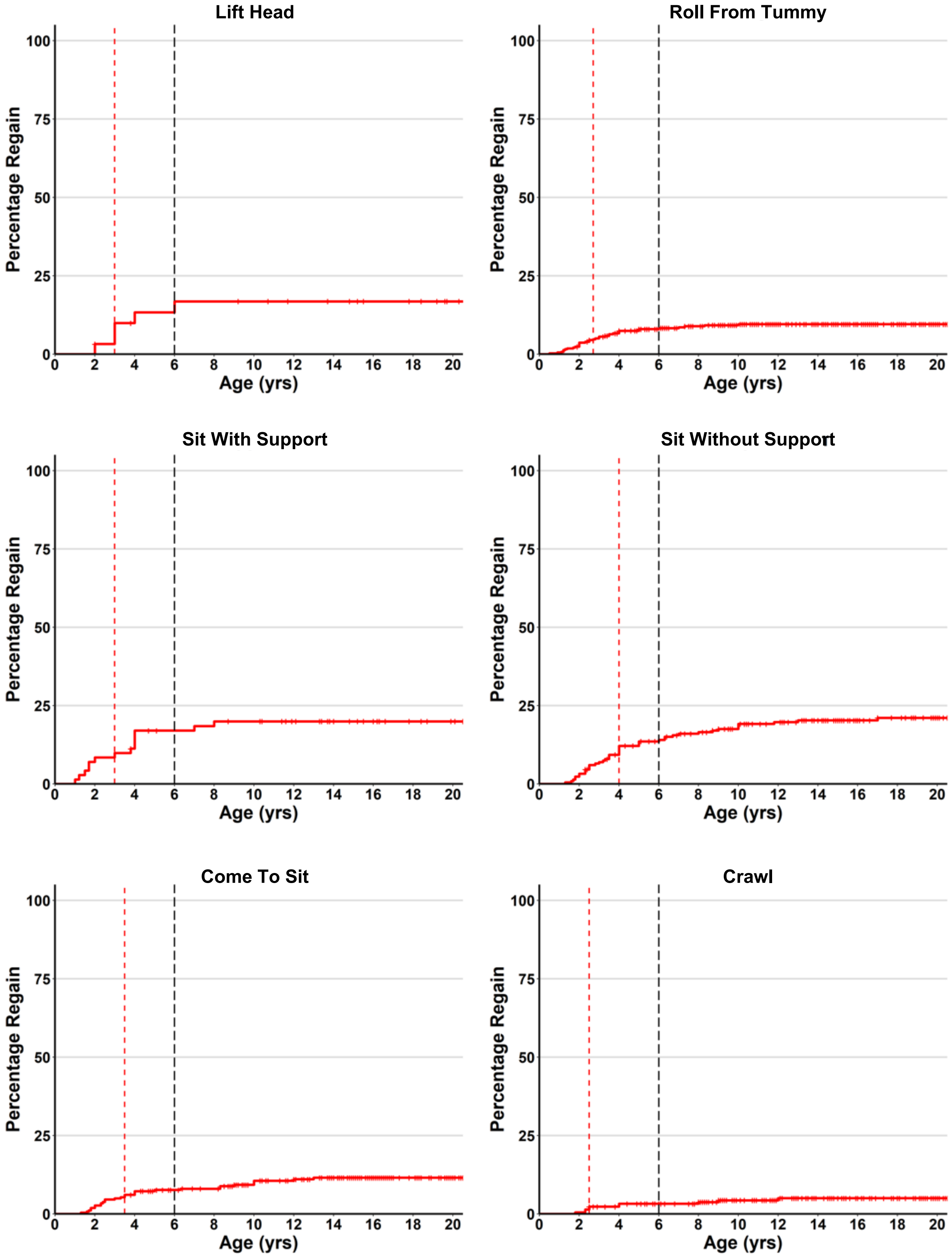

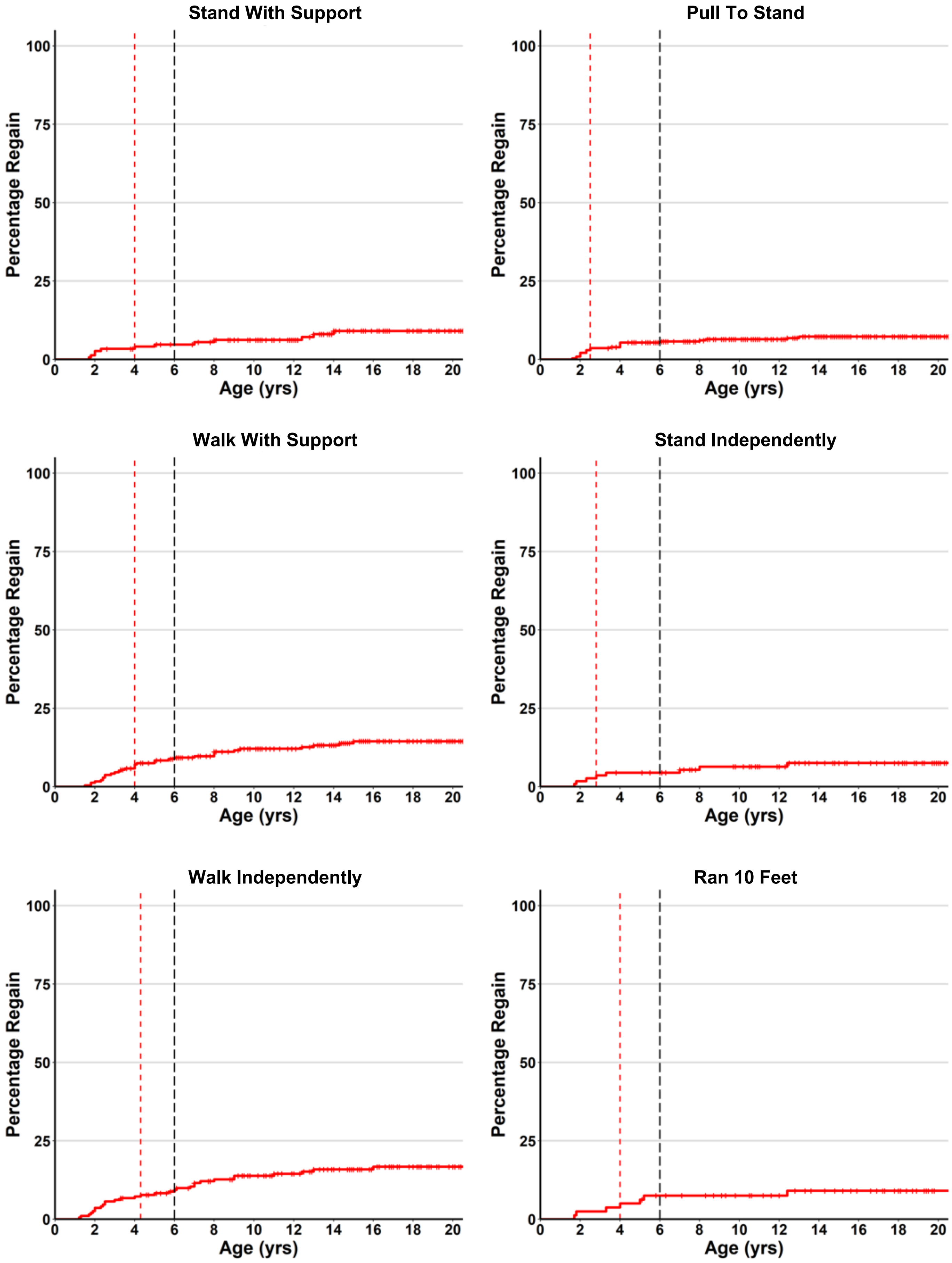
Stand With Support
Pull To Stand
Walk With Support
Stand Independently
Walk Independently
Ran 10 Feet
Percentage Regain
Age (yrs)
0
25
50
75
100
0
2
4
6
8
10
12
14
16
18
20

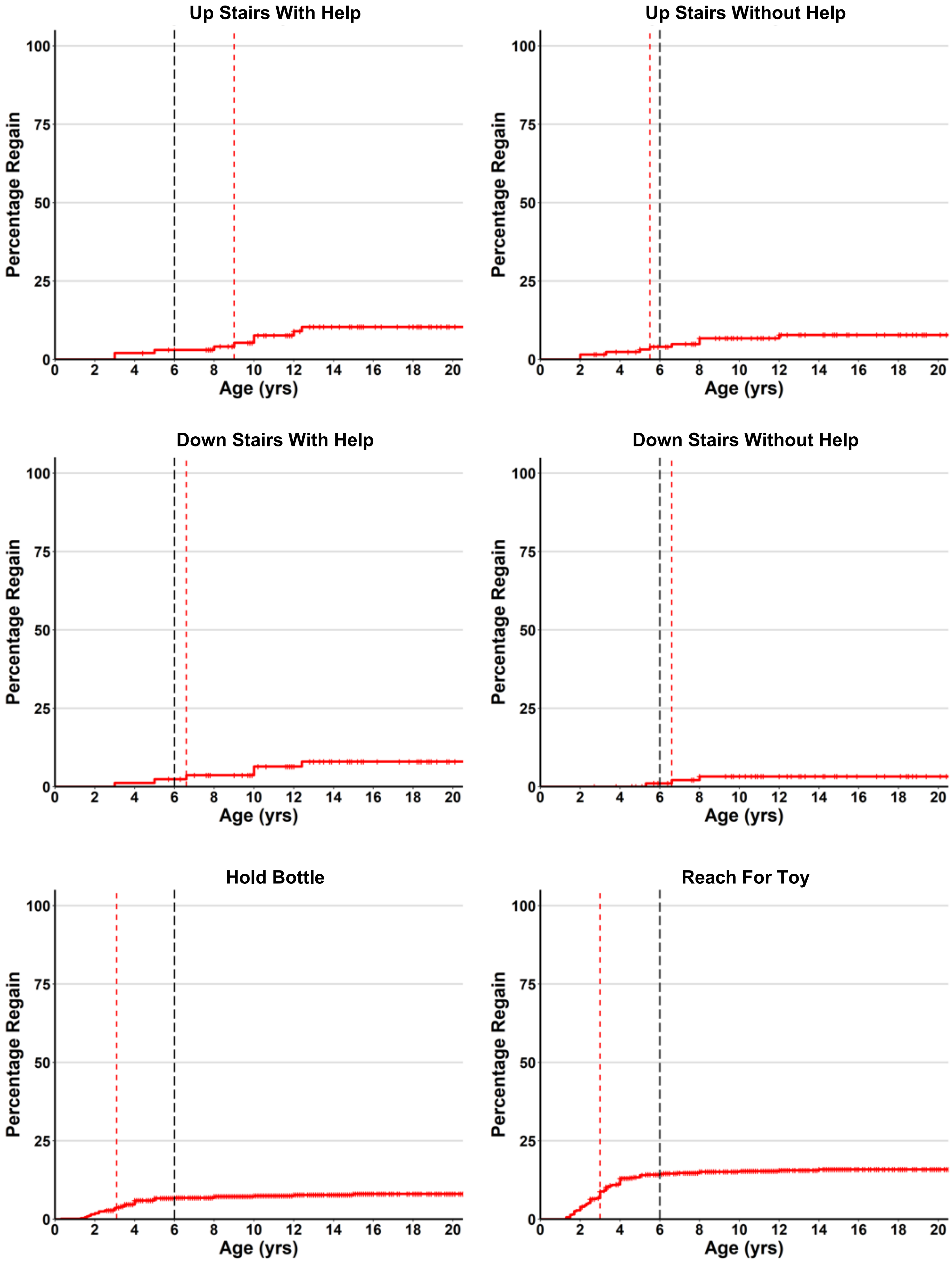

Up Stairs With Help
Up Stairs Without Help
Down Stairs With Help
Down Stairs Without Help
Hold Bottle
Reach For Toy
Percentage Regain
Age (yrs)
0
25
50
75
100
0 2 4 6 8 10 12 14 16 18 20

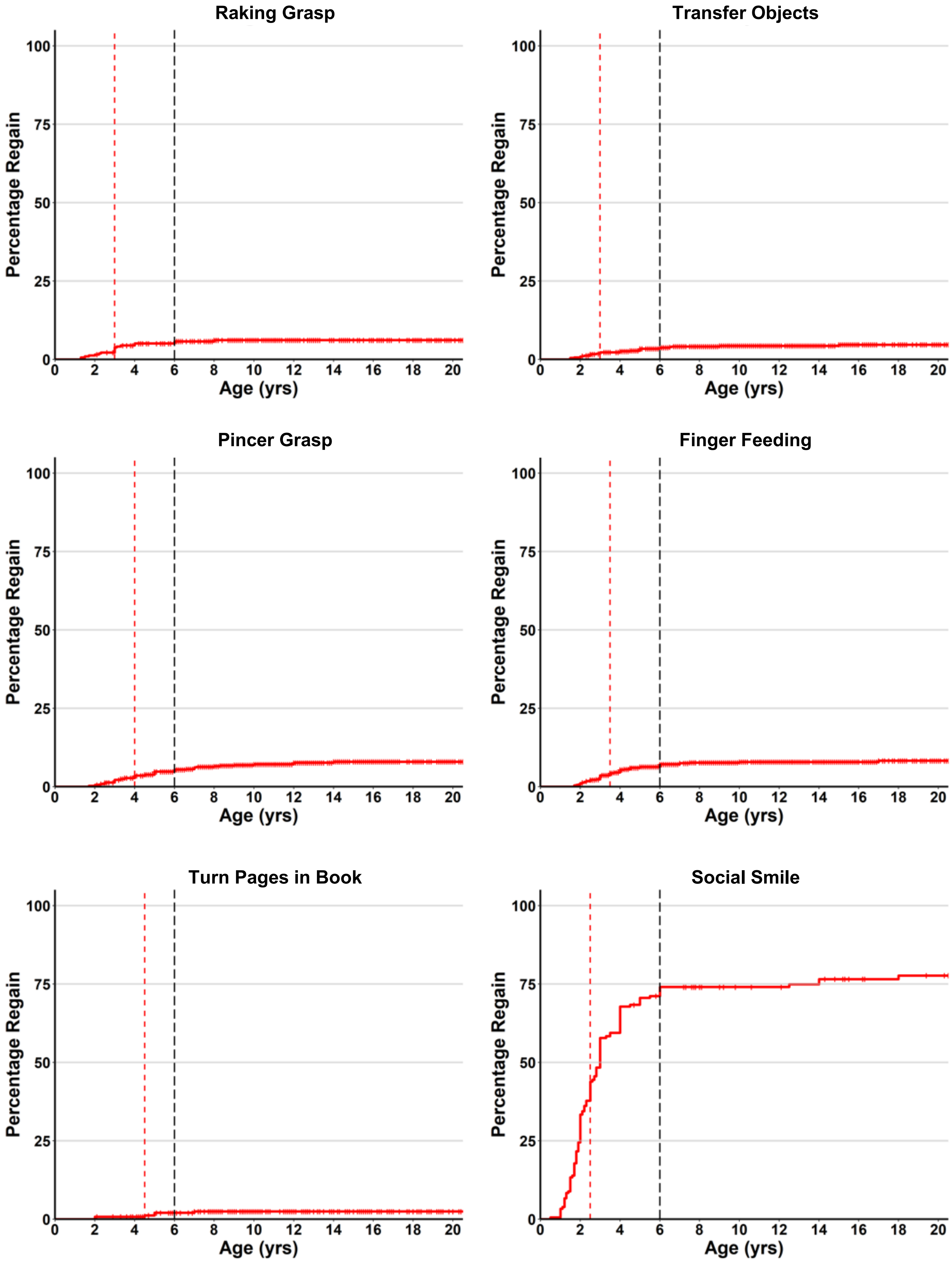

Raking Grasp
Transfer Objects
Pincer Grasp
Finger Feeding
Turn Pages in Book
Social Smile
Percentage Regain
Age (yrs)
0
25
50
75
100
0
2
4
6
8
10
12
14
16
18
20

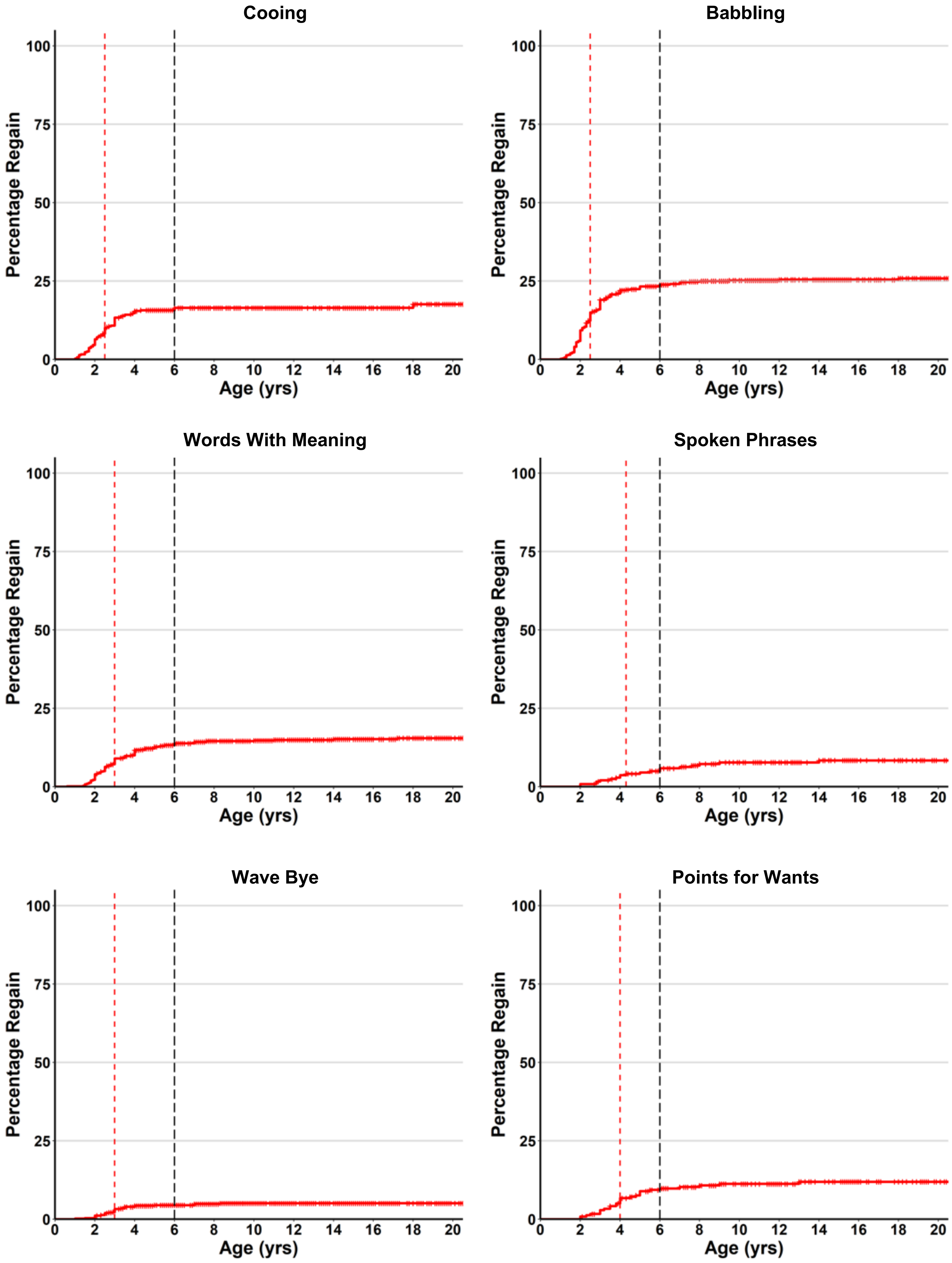
Cooing
Babbling
Words With Meaning
Spoken Phrases
Wave Bye
Points for Wants
Percentage Regain
Age (yrs)
0
25
50
75
100
0
2
4
6
8
10
12
14
16
18
20

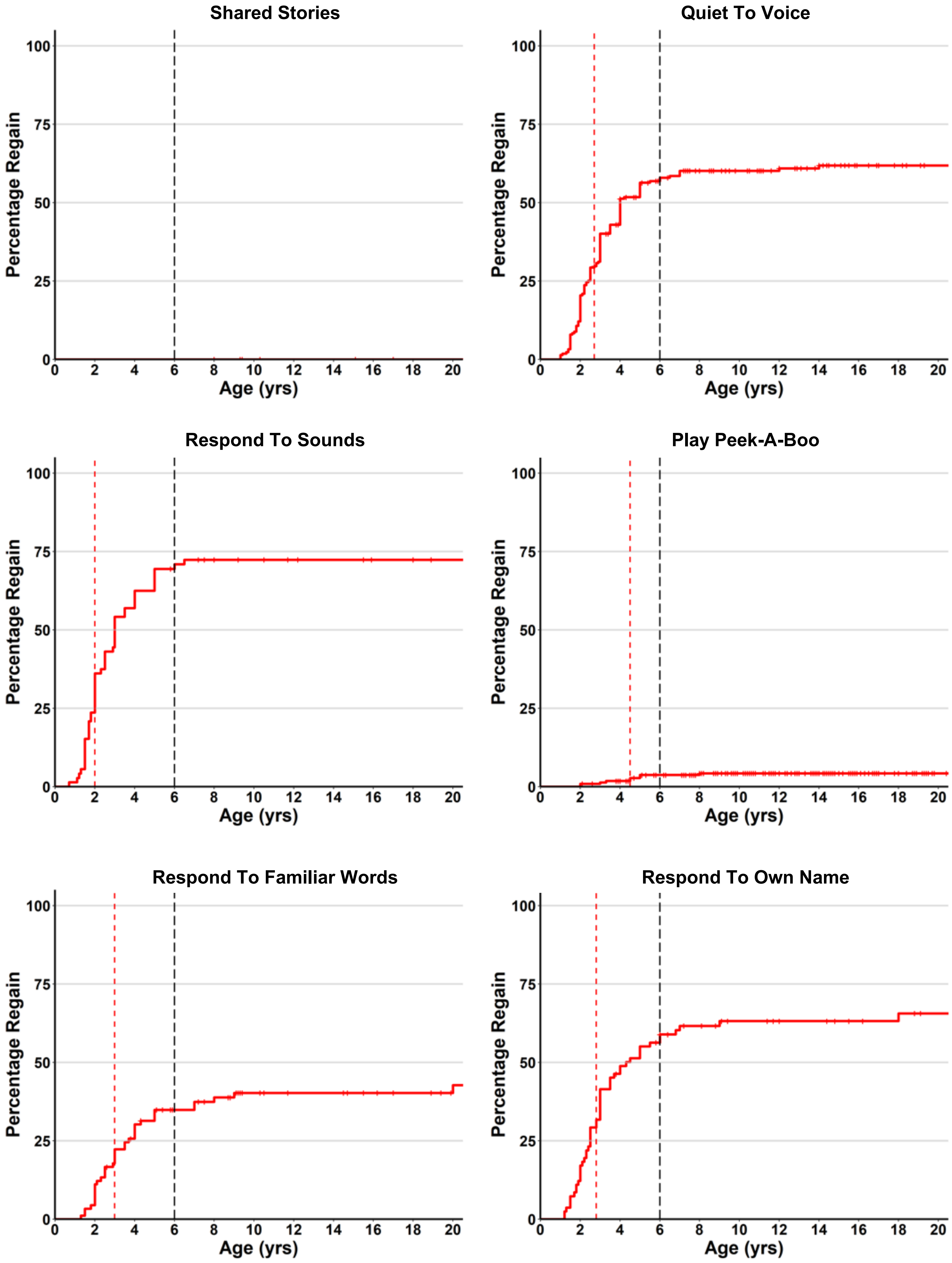

Shared Stories
Quiet To Voice
Respond To Sounds
Play Peek-A-Boo
Respond To Familiar Words
Respond To Own Name
Percentage Regain
0
25
50
75
100
Age (yrs)
0
2
4
6
8
10
12
14
16
18
20

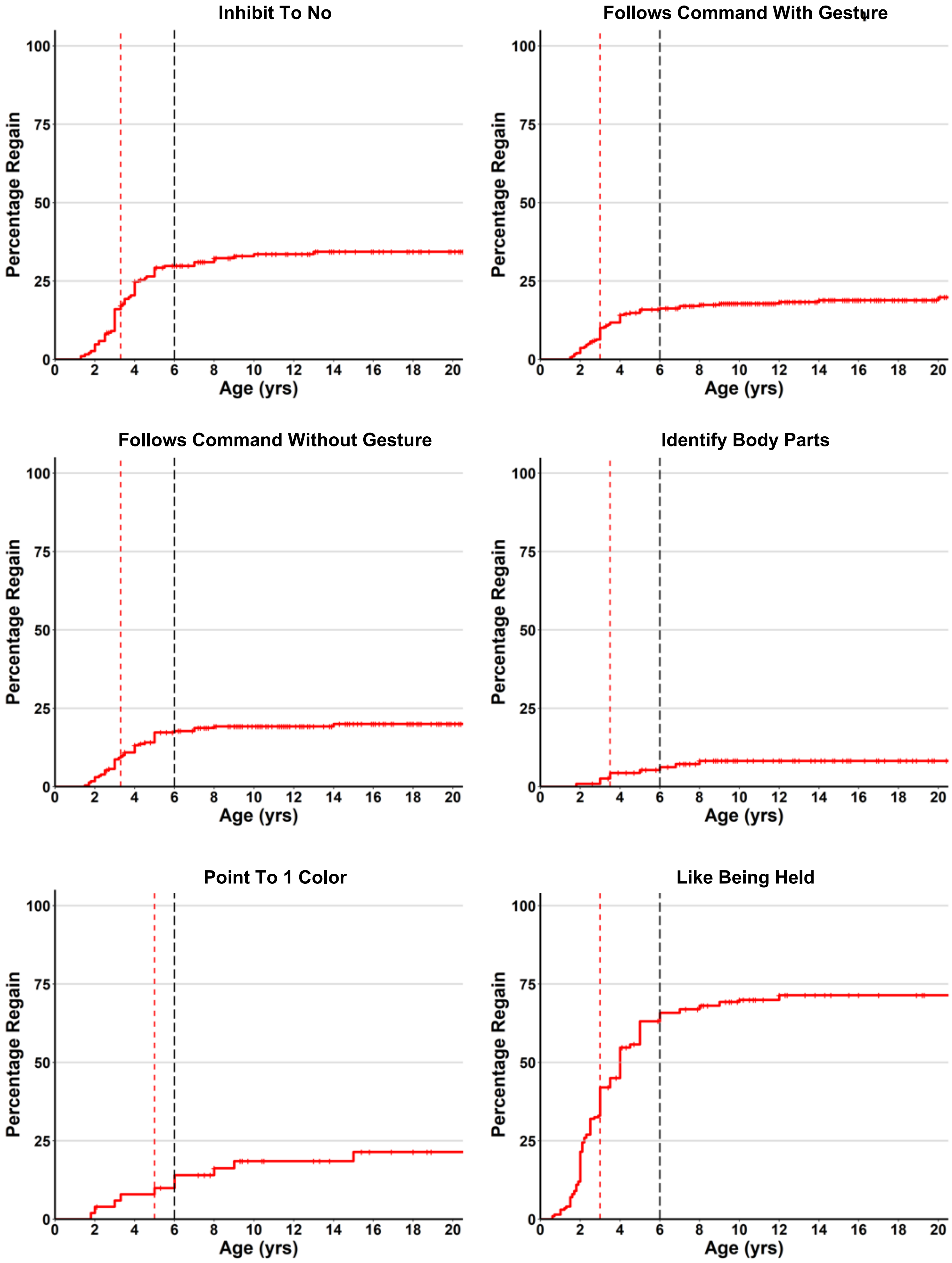

Inhibit To No
Follows Command With Gesture
Follows Command Without Gesture
Identify Body Parts
Point To 1 Color
Like Being Held
Percentage Regain
0
25
50
75
100
Age (yrs)
0 2 4 6 8 10 12 14 16 18 20

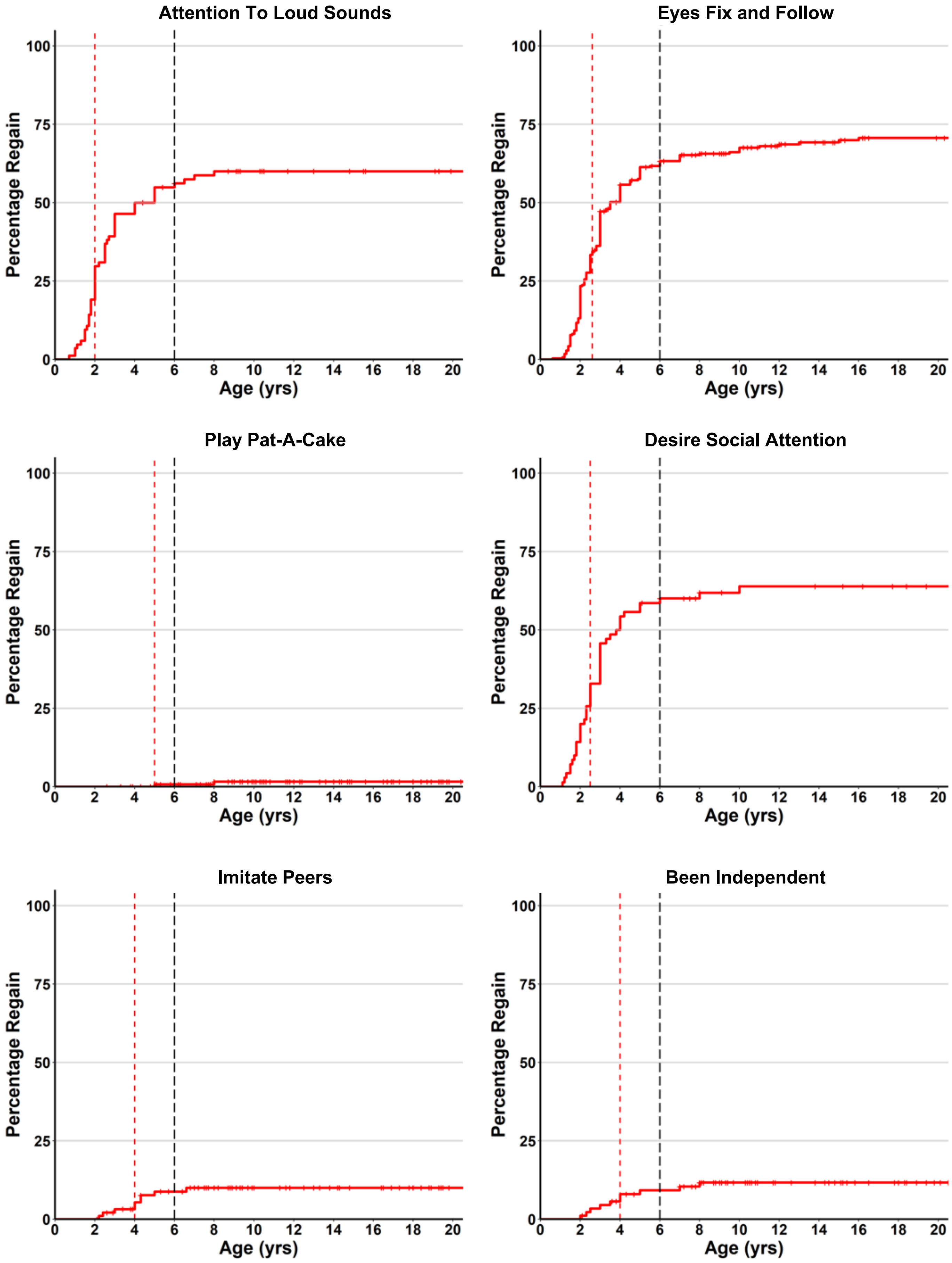
Attention To Loud Sounds
Eyes Fix and Follow
Play Pat-A-Cake
Desire Social Attention
Imitate Peers
Been Independent
Percentage Regain
Age (yrs)
0
25
50
75
100
0
2
4
6
8
10
12
14
16
18
20

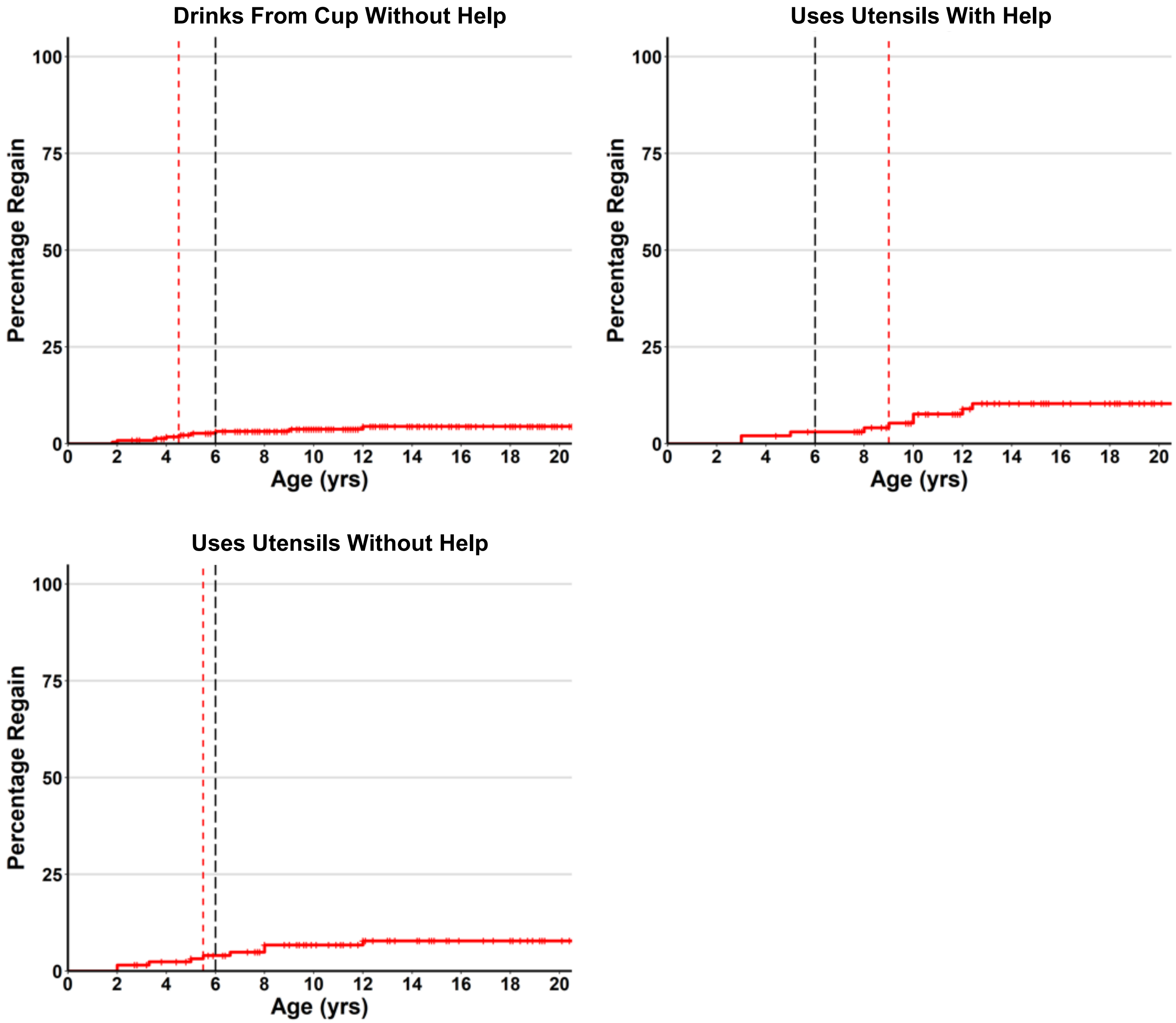

Drinks From Cup Without Help
Percentage Regain
Age (yrs)
Uses Utensils With Help
Percentage Regain
Age (yrs)
Uses Utensils Without Help
Percentage Regain
Age (yrs)
