# Supplementary material for: Trajectory of skill acquisition, loss, and regain in females with classic Rett syndrome
Source: J Neurodev Disord. 2026 Mar 12;18:20. doi: 10.1186/s11689-026-09680-6 (PMC13094048; doi:10.1186/s11689-026-09680-6)
Supplement: Supplementary file 8 — Supplementary Material 8 [file 11689_2026_9680_MOESM8_ESM.pdf]

Figure S5: Cumulative incidence curves of time from skill loss to regain. Censored data points are shown as cross lines. The red vertical line is the median time from loss to regain for that specific skill, with the black dashed line demarks 2 years from loss to regain.

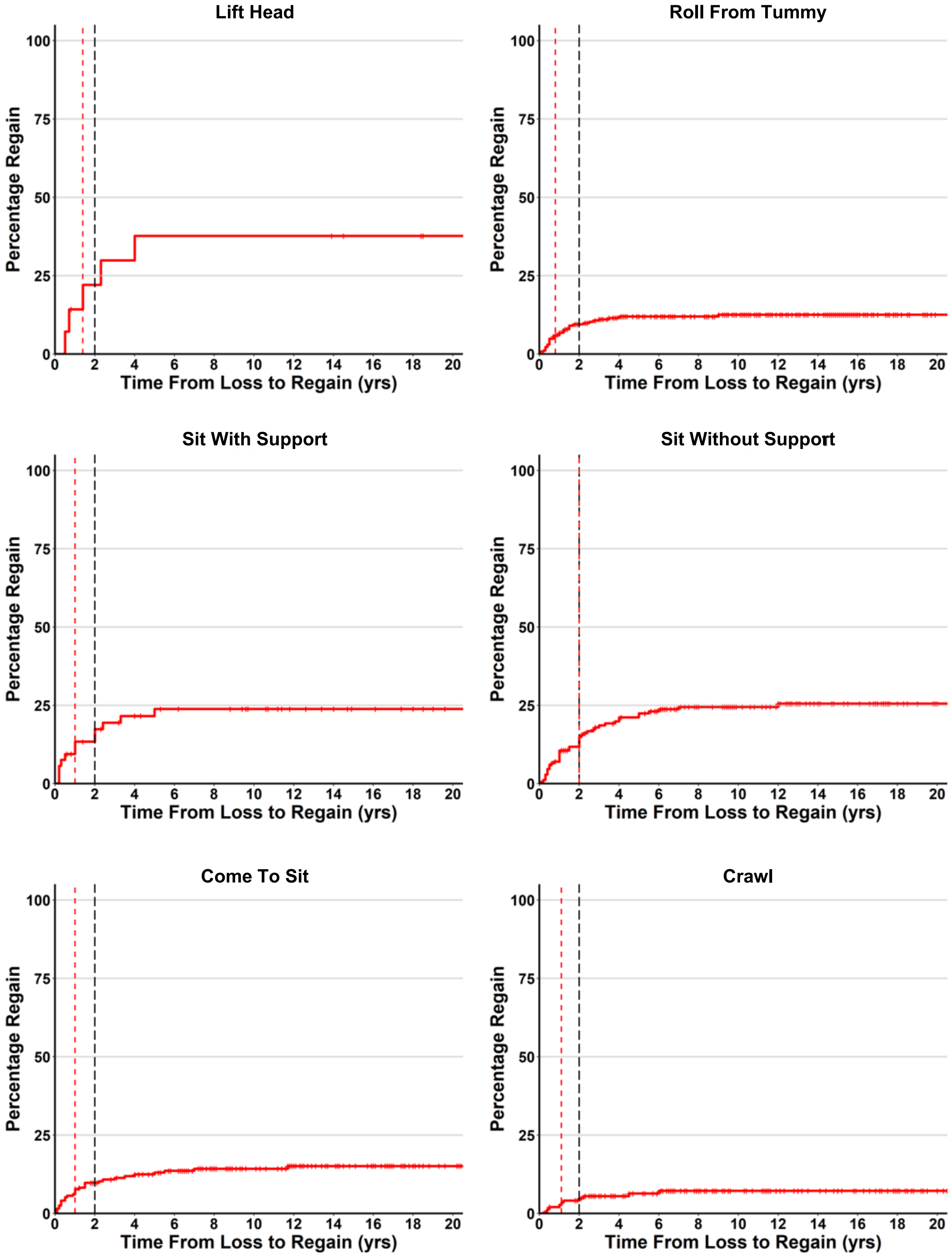

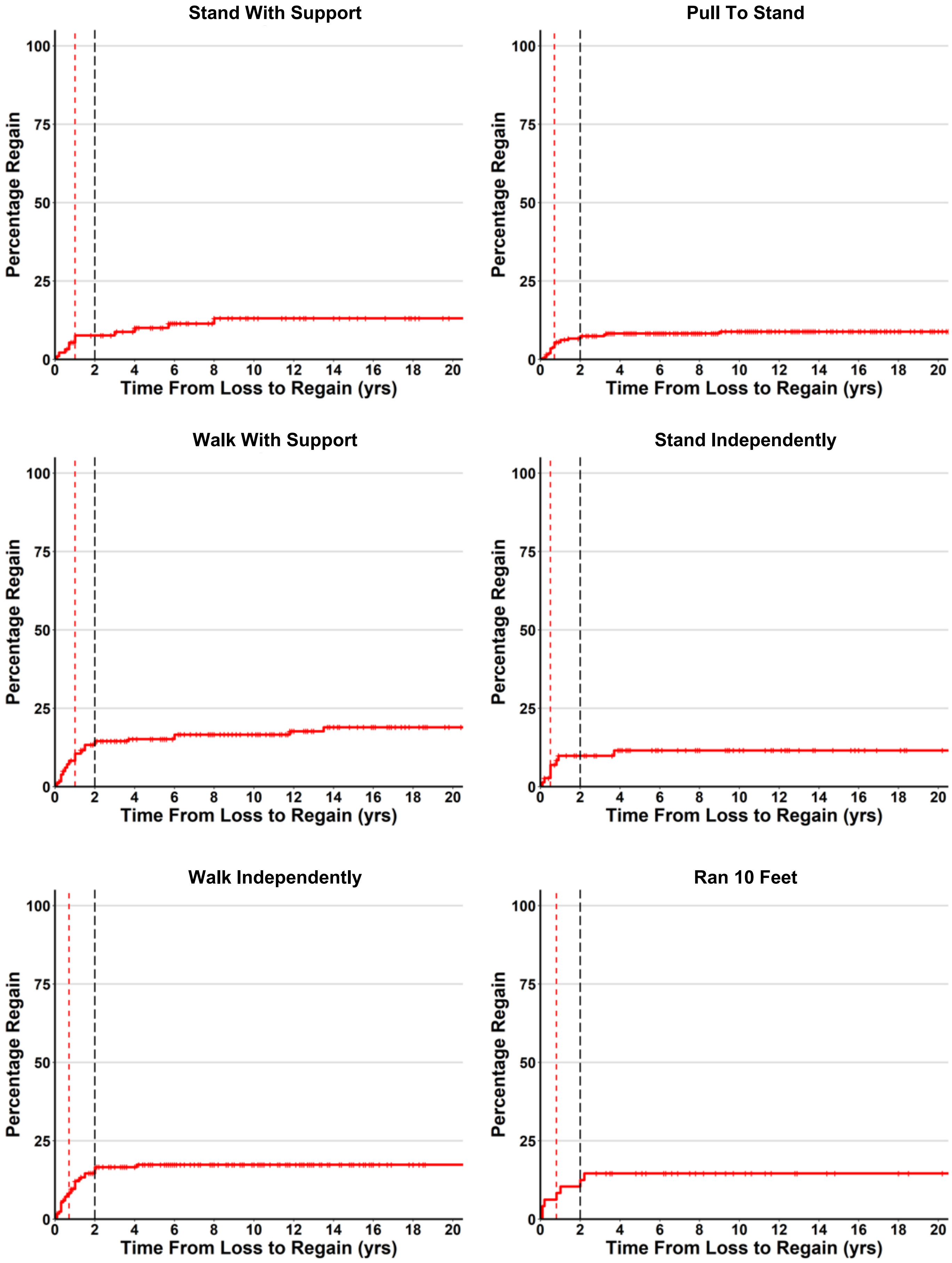
Stand With Support
Pull To Stand
Walk With Support
Stand Independently
Walk Independently
Ran 10 Feet
Percentage Regain
Time From Loss to Regain (yrs)
0
25
50
75
100
0
2
4
6
8
10
12
14
16
18
20

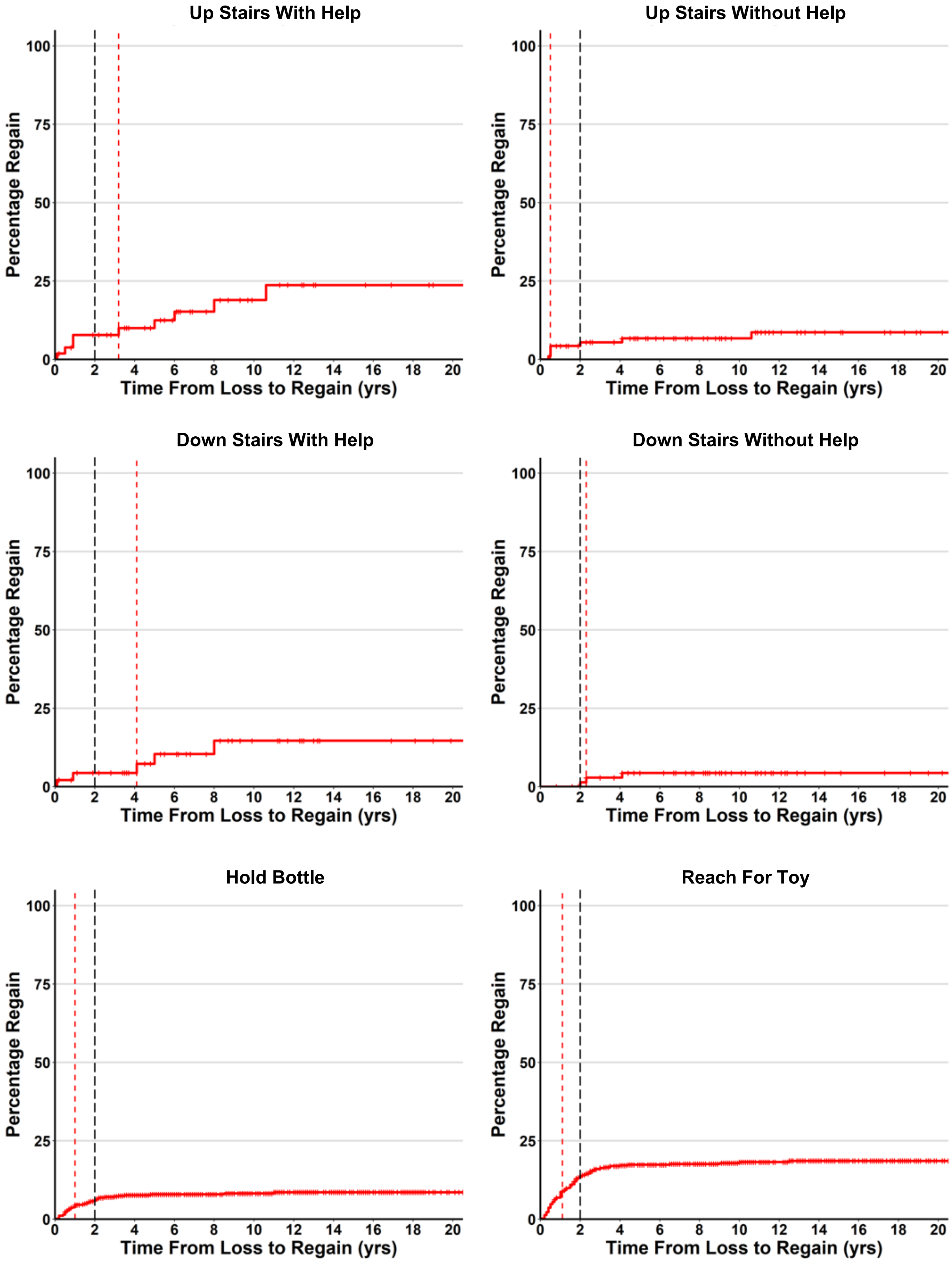

Up Stairs With Help
Up Stairs Without Help
Down Stairs With Help
Down Stairs Without Help
Hold Bottle
Reach For Toy
Percentage Regain
Time From Loss to Regain (yrs)
0
25
50
75
100
0
2
4
6
8
10
12
14
16
18
20

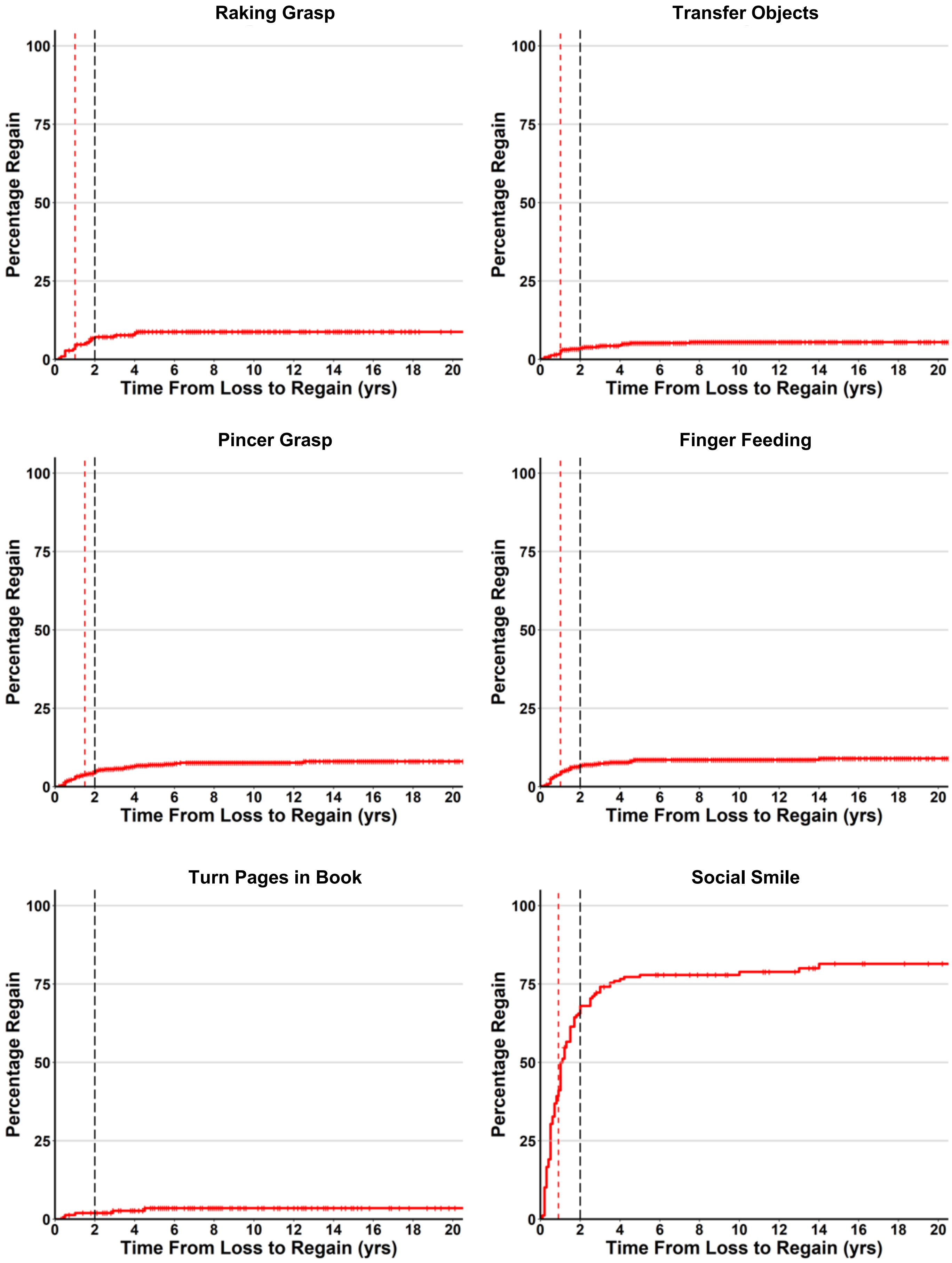

Raking Grasp
Transfer Objects
Pincer Grasp
Finger Feeding
Turn Pages in Book
Social Smile
Percentage Regain
0
25
50
75
100
Time From Loss to Regain (yrs)
0
2
4
6
8
10
12
14
16
18
20

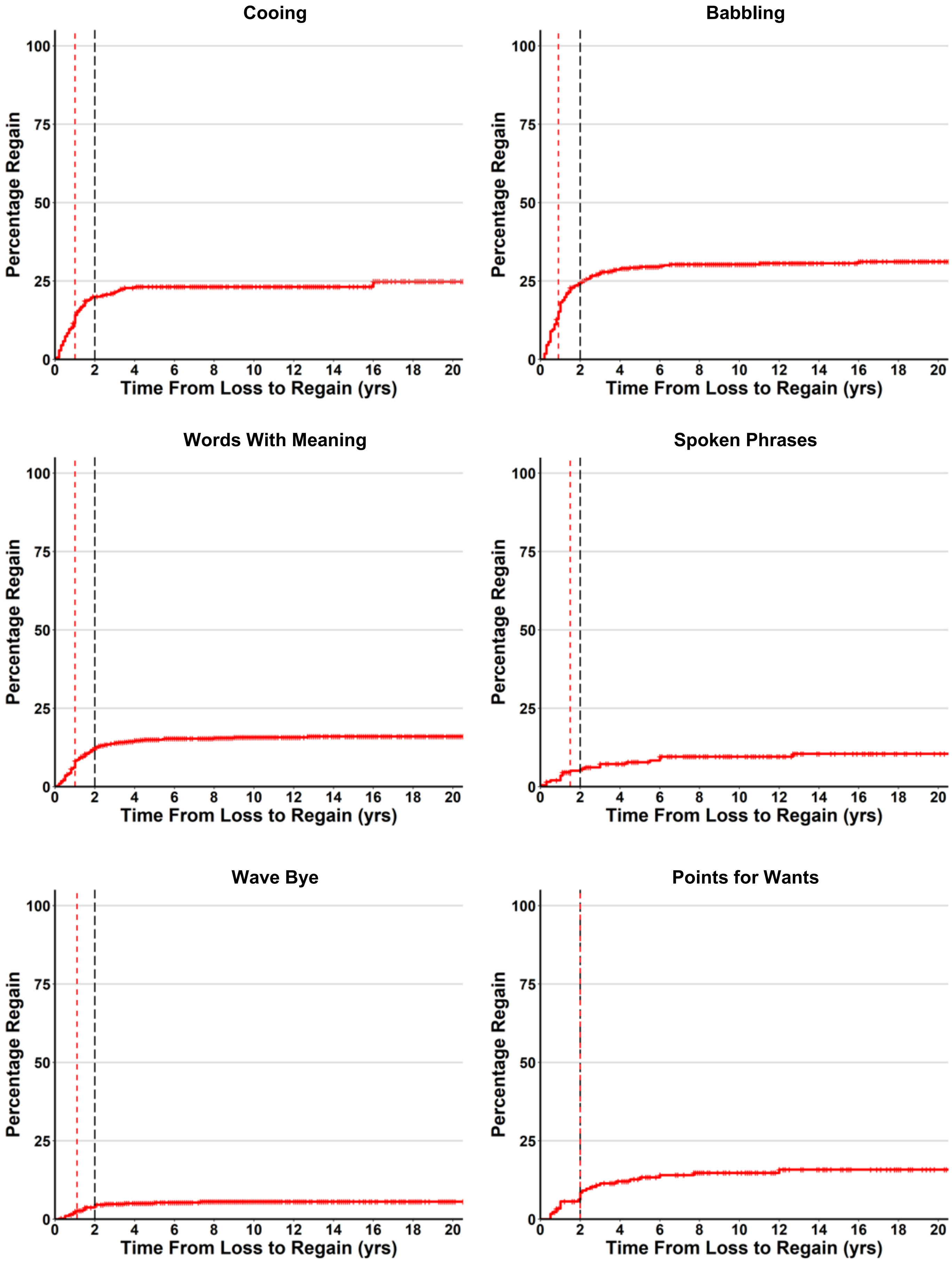
Cooing
Babbling
Words With Meaning
Spoken Phrases
Wave Bye
Points for Wants
Percentage Regain
Time From Loss to Regain (yrs)
0
25
50
75
100
0
2
4
6
8
10
12
14
16
18
20

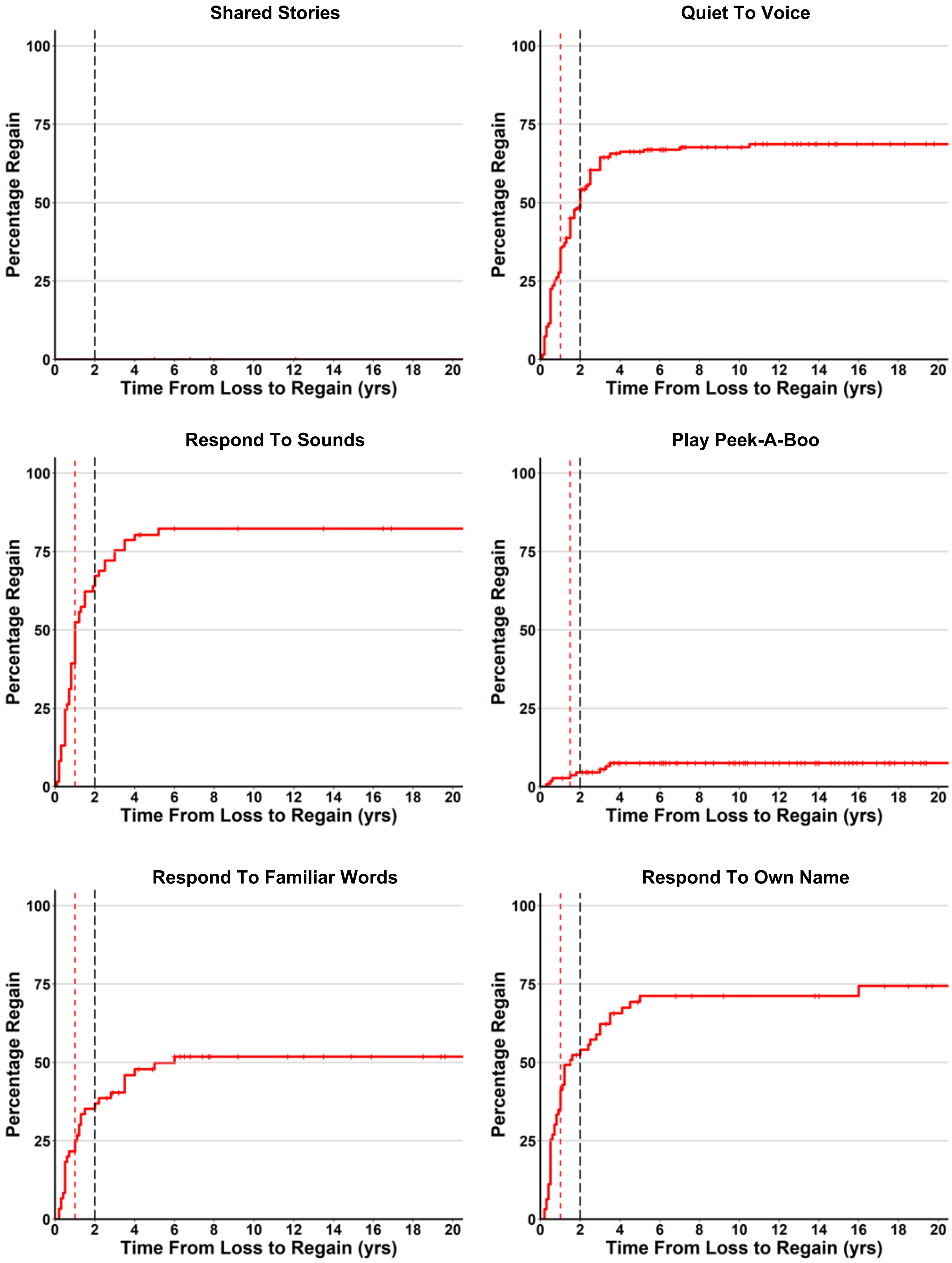

Shared Stories
Quiet To Voice
Respond To Sounds
Play Peek-A-Boo
Respond To Familiar Words
Respond To Own Name
Percentage Regain
Time From Loss to Regain (yrs)
0
25
50
75
100
0 2 4 6 8 10 12 14 16 18 20

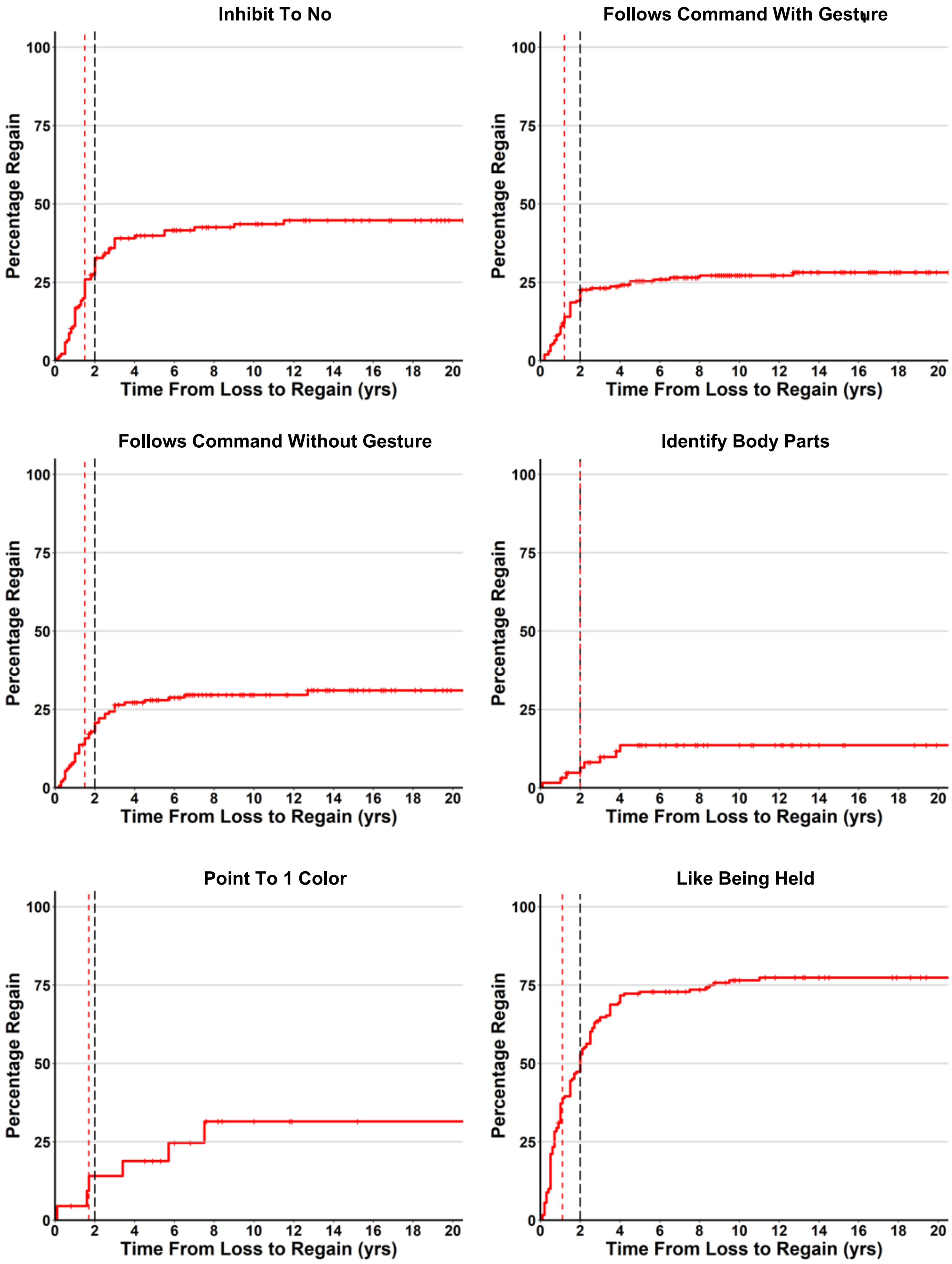

Inhibit To No
Follows Command With Gesture
Follows Command Without Gesture
Identify Body Parts
Point To 1 Color
Like Being Held
Percentage Regain
0
25
50
75
100
Time From Loss to Regain (yrs)
0
2
4
6
8
10
12
14
16
18
20

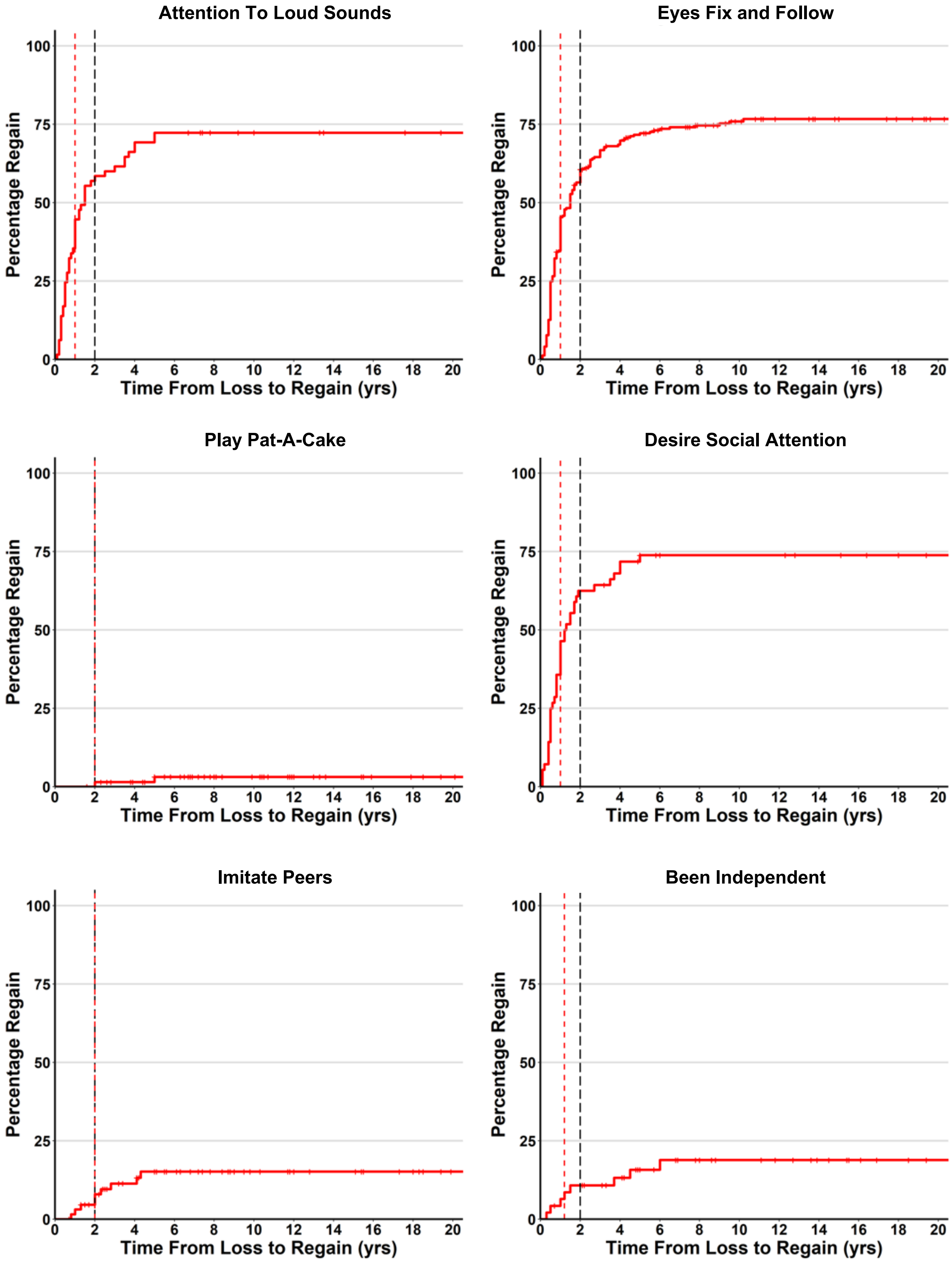
Attention To Loud Sounds
Eyes Fix and Follow
Play Pat-A-Cake
Desire Social Attention
Imitate Peers
Been Independent
Percentage Regain
Time From Loss to Regain (yrs)
0
25
50
75
100
0
2
4
6
8
10
12
14
16
18
20

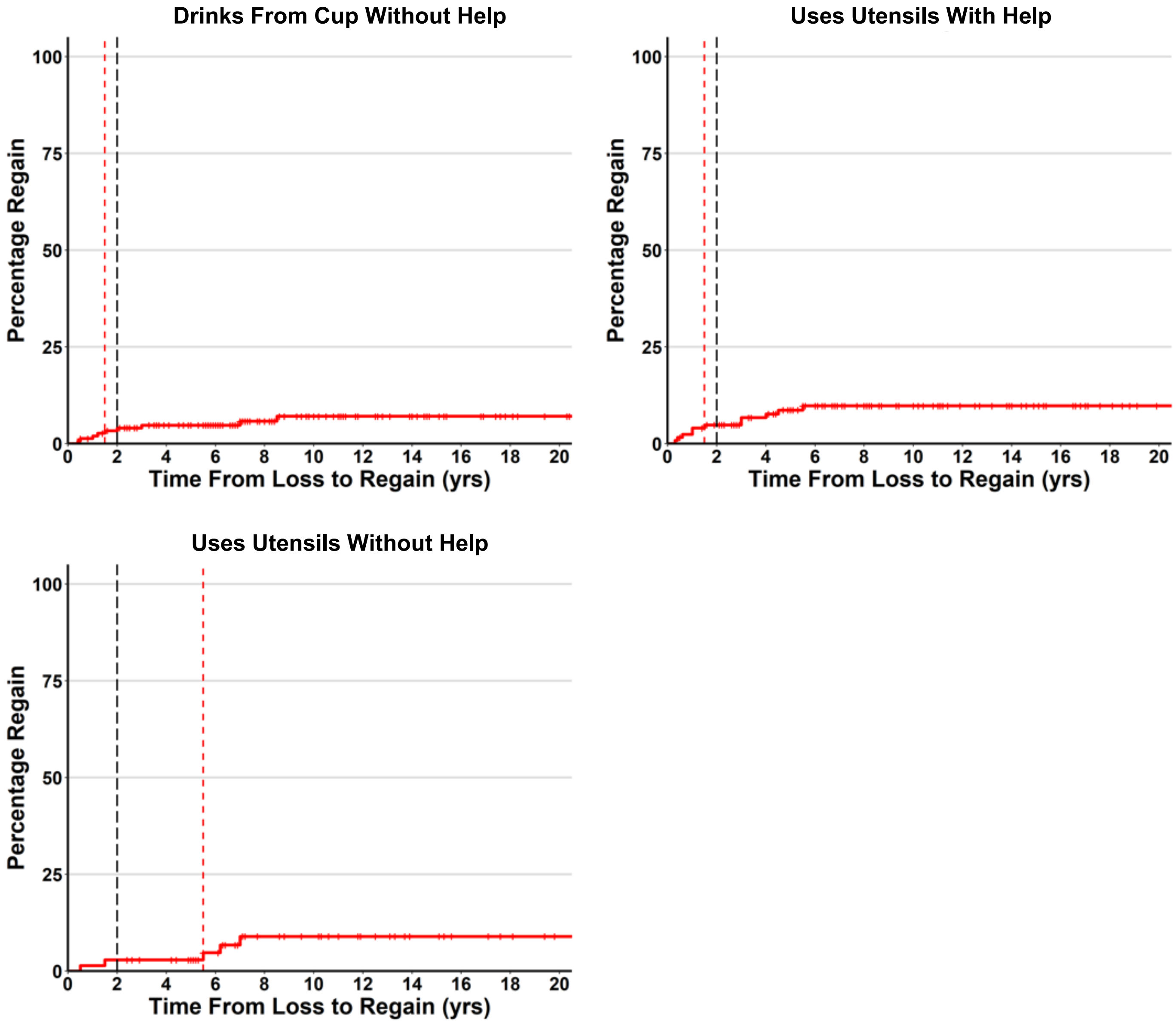

Drinks From Cup Without Help
Percentage Regain
100
75
50
25
0
0
2
4
6
8
10
12
14
16
18
20
Time From Loss to Regain (yrs)
Uses Utensils With Help
Percentage Regain
100
75
50
25
0
0
2
4
6
8
10
12
14
16
18
20
Time From Loss to Regain (yrs)
Uses Utensils Without Help
Percentage Regain
100
75
50
25
0
0
2
4
6
8
10
12
14
16
18
20
Time From Loss to Regain (yrs)
