# Supplementary material for: Trajectory of skill acquisition, loss, and regain in females with classic Rett syndrome
Source: J Neurodev Disord. 2026 Mar 12;18:20. doi: 10.1186/s11689-026-09680-6 (PMC13094048; doi:10.1186/s11689-026-09680-6)
Supplement: Supplementary file 9 — Supplementary Material 9 [file 11689_2026_9680_MOESM9_ESM.pdf]

SUPPLEMENTAL METHODS

For each skill, there were three data fields for gain, loss, and regain as follows, with X indicating Gain (G), Loss (L), or Regain (R):

E_X=Skill Event Occurrence (Yes/No/Blank)
T_X=Age of Skill Occurrence if known (months or Blank)
U_X=Age of Skill Occurrence Unknown (Yes or Blank)

Thus, for each of the 51 skills, there are 9 data fields for each participant (total of 459 data fields for skills). For 1228 participants, there are a total of 563,652 cells (1228 x 459) for developmental skill data.

For each participant, there is also the age of last visit:

T_F=Age of Last Visit

Data were cleaned prior to analysis according to the following set of rules. Prior to cleaning, data were re-coded by changing "Yes" entries to "1" and "No" entries to "0".

Data Cleaning Process:

1. Changes to skill event occurrence data cells:
    a. If preceding skill event did not occur and the subsequent skill event also had an indicator that it did not occur, then the subsequent skill event indicator was changed to blank.
        i. (IF E_G=0 AND E_L=0, THEN E_L= Blank) OR (IF E_L=0 AND E_R=0, THEN E_R=Blank)
        ii. Total number of changes = 22,536 (4.0% all data cells)
    b. If preceding skill event occurred and subsequent indicator was blank, then subsequent skill event indicator changed to "No"
        i. (IF E_G=1 AND E_L=Blank, THEN E_L= 0) OR (IF E_L=1 AND E_R=Blank, THEN E_R=0)
        ii. Total number of changes = 852 (0.15% all data cells)
    c. If preceding skill event indicator said event did not occur but subsequent event did occur, then the preceding event indicator was changed to "Yes" and age of event indicated as "Unknown".
        i. (IF E_G=0 AND E_L=1, THEN E_G=1 AND U_G=1) OR (IF E_L=0 AND E_R=1, THEN E_L=1 AND U_L=1)
        ii. Total number of changes = 255 (0.05% all data cells)
    d. If skill event indicator was blank but age of event or age of event unknown indicated, changed event indicator to "Yes"
        i. (IF E_G=Blank AND either T_G=value OR U_G=1, THEN E_G=1) OR (IF E_L=Blank AND either T_L=value OR U_L=1, THEN E_L=1) OR (IF E_R=Blank AND either T_R=value OR U_R=1, THEN E_R=1)
        ii. Total number of changes = 114 (0.02% all data cells)
    e. **Total changes to event occurrence = 23,757 (4.22% of all data cells)**
2. Changes to age of event unknown indicator:
    a. If skill event occurred but both the age of event and the age of event unknown indicator blank, then changed age of event unknown indicator to "Yes"
        i. (IF E_G=1 AND T_G=Blank AND U_G=Blank, THEN U_G=1) OR (IF E_L=1 AND T_L=BLANK AND U_L=Blank, THEN U_L=1) OR (IF E_R=1 AND T_R=Blank AND U_R=Blank, THEN U_R=1)
    b. If event occurred AND age of event indicated AND age of event unknown, then the age of event unknown indicator was changed to "Blank"

            i. (IF E_G=1 AND T_G=value AND U_G=1, THEN U_G=Blank) OR (IF E_L=1 AND T_L=value AND U_L=1, THEN U_L=Blank) OR (IF E_R=1 AND T_R=value AND U_R=1, THEN U_R=Blank)

    c. **Total changes to age of event unknown indicator = 1,205 (0.2% of all data cells)**

3. <u>Changes to age of event to correct logical inconsistencies:</u>
    a. If age of event occurred after age of last visit or occurred before age of preceding event, then the age of event was changed to "Blank" and the age of event unknown indicator changed to "Yes"
        i. (IF $T_G > T_F$, THEN T_G=Blank AND U_G=1) OR (IF $T_L > T_F$ OR $T_L < T_G$, THEN T_L=Blank AND U_L=1) OR (IF $T_R > T_F$ OR $T_R < T_L$, THEN T_R=Blank AND U_R=1)
    b. **Total changes to age of event = 736 (0.13% of all data cells)**
4. <u>Changes to age of gain based on entered values being unrealistically young:</u>
    a. The age of skill gain entered was unrealistically young and assumed to be a data entry error. For example, for 4 participants the age of gaining the ability to walk independently was entered as less than 4 months of age, which is less than the 1% cutoff for typically developing individuals from the 2006 WHO study (8.2 months [1]). We systematically applied lower age of gain cutoffs for 39 skills (see table below for skills and age of gain cutoffs for each skill). The ages of gain cutoffs were defined based on published normative data [2] with a conservative approach (i.e. generally well below the lower limit from normative data).
    b. For cases meeting these specified criteria, the age of gain was changed to "Blank" and the age of gain unknown changed to "Yes"
        i. (IF T_G<=lower age cutoff, THEN T_G=Blank AND U_G=1)
    c. **Total changes for unrealistic age of gain: 590 (0.1% of all data cells)**
5. <u>Changes made based on manual review of source data:</u>
    a. For two participants with data from both 5201 and 5211 protocol, there were discrepancies in the age of skill event for a single skill (Come to Sitting Gain Age for one participant, Walk Independently Regain Age for one participant). The age of skill event was changed to match the values in 5201 (which was collected first).
    b. For one participant, the age of regain for Walk Independently was identified as an outlier (252 months, 21 years old). Source data was reviewed and both caregiver report and clinical assessment indicated that skill (Walk Independently) was not present. Regain occurrence changed to "No", age of regain changed to "Blank. (E_R=0 AND T_R=Blank).
    c. Two participants had a number of late skill losses and late skill regains. Manual review of source data indicated that for both participants, these late skill losses and late skill regains were associated with scoliosis and other orthopedic surgical procedures and subsequent repeat corrective orthopedic surgeries for failed previous surgeries, or gall bladder surgeries. For these skill losses or regain, the age of event was changed to "Blank" and age unknown for event changed to "Yes". (T_L=Blank AND U_L=1 OR T_R=Blank AND U_R=1)
    d. **Total number changes based on review of source data = 64 (0.01% of all data cells)**
6. <u>Summary of changes made based on all above criteria:</u>
    a. **N=26,170 (4.64% of all data cells)**
        i. The majority of changes were to event occurrence indicators (n=23,575, 4.22%), with the vast majority of these removing subsequent event indicator of "No" when preceding event did not occur (n=22,537, 4.0%). The remaining changes to event occurrence indicators (n=1,221, 0.22%) added missing indicators to subsequent events when preceding event occurred, correcting preceding event indicators when subsequent

indicator occurred, or adding missing event indicator when age of event provided or age of event unknown was indicated.

ii. The next largest group of changes were corrections to event unknown indicator to remove event unknown indicator when age of event provided, or add event unknown indicator when event occurred but age of event not entered and age of event unknown indicator blank (n=1,205, 0.2%)
iii. Correcting logical age inconsistencies (age event after age of last visit or age event before preceding event age) was the next largest group of changes (n=736, 0.13%)
iv. Identification of unrealistically young age of gain and removing age of gain and adding age of gain unknown indicator was the next largest group of changes (n=590, 0.1%)
v. Finally, the smallest group of changes were based on review of source data (n=64, 0.01%)

Table of ages of skill gain identified as unrealistically young.

Skill	Age cutoff (months) for unrealistic age of gain
Sit With Support	2
Sit Without Support	3
Come To Sit	2
Crawl	3
Stand With Support	3
Pull To Stand	4
Walk With Support	5
Stand Independently	4
Walk Independently	4
Ran 10 Feet	8
Up Stairs With Help	5
Up Stairs Without Help	7
Down Stairs With Help	2
Down Stairs Without Help	5
Reach For Toy	1
Raking Grasp	3
Transfer Objects	2
Finger Feeding	2
Turn Pages in Book	3
Babbling	1.5
Words With Meaning	5
Spoken Phrases	11
Wave Bye	4
Point for Wants	1
Shared Stories	12
Play Peek-A-Boo	2
Respond To Familiar Words	2
Respond To Own Name	3
Inhibit To No	2
Follows Commands With Gesture	4
Follows Commands Without Gesture	5
Identify Body Parts	7
Point to 1 Color	5
Play Pat-A-Cake	3
Imitate Peers	6
Been Independent	6
Drinks From Cup Without Help	5
Uses Utensils With Help	5
Uses Utensils Without Help	3

After cleaning and prior to analysis, all ages were converted from "months" to "years" (with 1 decimal point) by dividing months by 12. For all events that did not occur (E_X=0), the age of last visit (in years) was inserted into the age of event in order to include this information as "censored age" data for subsequent survival analysis.

<u>Calculation of one-year interval incidence of gain or regain:</u>
The incidence of either gain or regain of each skill during a one-year interval was calculated for each one-year interval from age 0-20 years old. Data beyond 20 years old was truncated and not included in this analysis. In every one-year interval, each participant was determined if they were eligible to gain or regain the specific skill, and if they did gain or regain that skill or not during that interval. The determination of eligibility for a given participant and specific skill within the interval and classification of having gained or regained the skill in the interval (coded as "1") or not (coded as "0"), as well as if they were not eligible to potentially gain or regain that skill within the interval (coded as "N/A") is diagramed within the figure below, which includes the definitions used and the logical expressions defining the conditions using Excel expressions. For each one-year interval, the percentage of individuals who gained or regained a skill was calculated as the ratio of the total number of participants who gained or regained the skill ("1" in logic diagram) divided by total number of eligible participants in the interval (those who gained or regained the skill ["1"] plus those eligible who did not gain or regain the skill ["0"]). Participants assigned a value of "#N/A" for a given skill in a specific interval were not included in the calculation for that interval.

Logic diagram used for calculation of one-year interval incidence of skill gain or regain

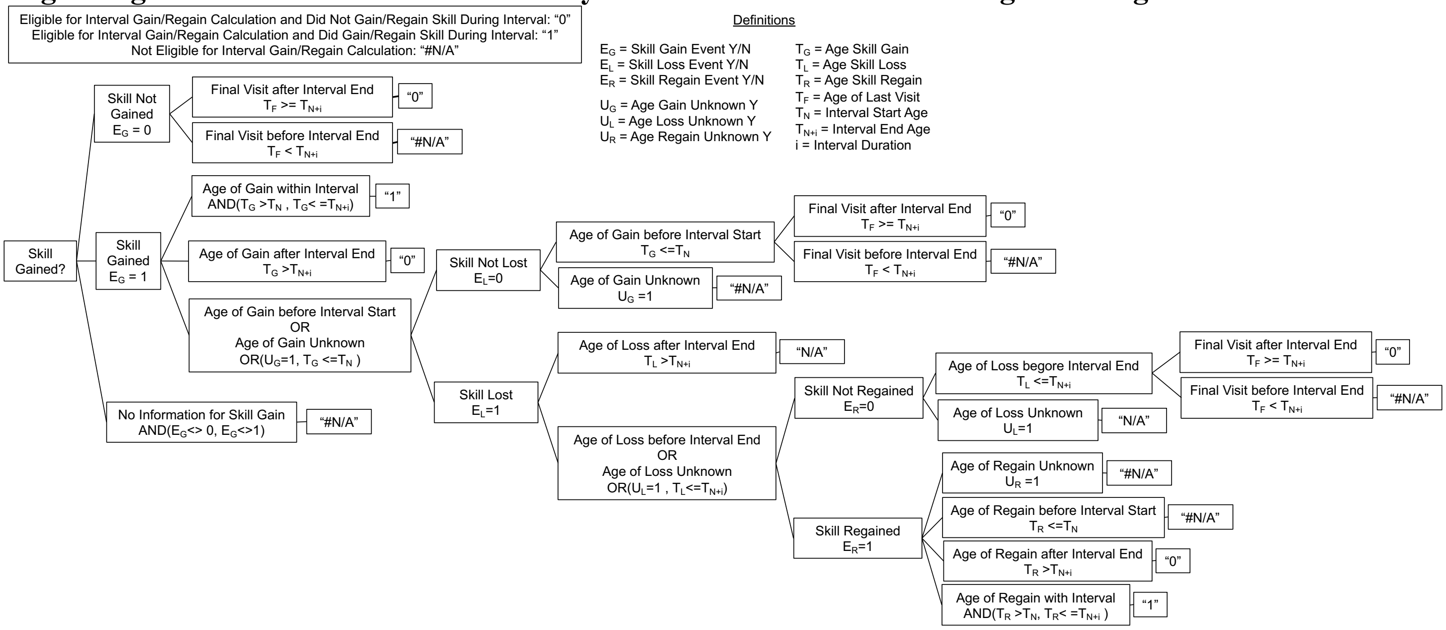

Below are the specific Excel functions used to classify the conditions, as well as the complete multiple if function used to assign a value for a skill for an individual in a specific interval.

<u>"1": Gain/Regain occurred during interval:</u>
$AND(E_G=1, OR(AND(U_G<>1, T_G> T_N, T_G<=T_{N+i}), AND(E_R=1, U_R<>1, T_R> T_N, T_R<=T_{N+i})))$

<u>"0": Gain/Regain did not occur during interval, but eligible (counted for total in denominator):</u>
$OR(AND(E_G = 0, T_F >= T_{N+i}), AND(E_G = 1, OR(AND(U_G <> 1, OR(T_G >T_{N+i}, AND(E_L=0, T_G <=T_N, T_F >= T_{N+i}))), AND(E_L=1, OR(U_G = 1, AND(U_G <> 1, T_G <=T_N)), OR(AND(E_R=0, T_F >= T_{N+i}, AND(U_L <> 1, T_L <=T_{N+i})), AND(E_R=1, U_R <> 1, T_R >T_{N+i}, OR(U_L = 1, AND(U_L <> 1, T_L <=T_{N+i}))))))))$

<u>#N/A: Not included in count:</u>

OR(AND(E_G<>0, E_G<>1), AND(E_G=0, T_F<T_{N+i}), AND(E_G = 1, OR(U_G = 1, AND(U_G <> 1, T_G <=T_N)), OR(AND(E_L=0, OR(U_G = 1, AND(U_G <> 1, T_G <=T_N, T_F < T_{N+i}))), AND(E_L=1, OR(AND(U_L <> 1, T_L > T_{N+i}), AND(OR(U_L = 1, AND(U_L <> 1, T_L <=T_{N+i})), OR(AND(E_R=0, OR(U_L = 1, T_F < T_{N+i})), AND(E_R=1, OR(U_R = 1, AND(U_R <> 1, T_R <=T_N))))))))))

Complete Merged Excel Function:
=IFS(AND(E_G=1, OR(AND(U_G<>1, T_G>T_N, T_G<=T_{N+i}), AND(E_R=1, U_R<>1, T_R> T_N, T_R<=T_{N+i}))), **1**, OR(AND(E_G=0, T_F>=T_{N+i}), AND(E_G=1, OR(AND(U_G<>1, OR(T_G>T_{N+i}, AND(E_L=0, T_G <=T_N, T_F>=T_{N+i}))), AND(E_L=1, OR(U_G=1, AND(U_G<>1, T_G<=T_N)), OR(AND(E_R=0, T_F>=T_{N+i}, AND(U_L<>1, T_L<=T_{N+i})), AND(E_R=1, U_R<>1, T_R>T_{N+i}, OR(U_L=1, AND(U_L<>1, T_L<=T_{N+i})))))))), **0**, OR(AND(E_G<>0, E_G<>1), AND(E_G=0, T_F<T_{N+i}), AND(E_G=1, OR(U_G=1, AND(U_G<>1, T_G<=T_N)), OR(AND(E_L=0, OR(U_G=1, AND(U_G<>1, T_G<=T_N, T_F<T_{N+i}))), AND(E_L=1, OR(AND(U_L<>1, T_L>T_{N+i}), AND(OR(U_L=1, AND(U_L<>1, T_L<=T_{N+i})), OR(AND(E_R=0, OR(U_L=1, T_F<T_{N+i})), AND(E_R=1, OR(U_R=1, AND(U_R<>1, T_R<=T_N)))))))))), **#N/A**)

References

1. Group WHOMGRS. WHO Motor Development Study: windows of achievement for six gross motor development milestones. Acta Paediatr Suppl. 2006;450:86–95.
2. Sheldrick RC, Schlichting LE, Berger B, Clyne A, Ni P, Perrin EC, et al. Establishing New Norms for Developmental Milestones. Pediatrics. 2019;144(6).
